# R-spondin 3 is a myokine that mediates type I fiber determination during skeletal muscle regeneration

**DOI:** 10.1007/s11033-025-11099-6

**Published:** 2025-10-09

**Authors:** Teng Hu, Yoshitaka Mita, Xinhe Zhu, Yasuro Furuichi, Yoshimi Nakagawa, Yasuko Manabe, Nobuharu L. Fujii

**Affiliations:** 1 Department of Health Promotion Sciences, Graduate School of Human Health Sciences, Tokyo Metropolitan University, 1-1 Minami-Osawa, Hachioji, Tokyo Japan; 2 Department of Immunology, Graduate School of Medicine, Juntendo University, 2-1-1 Hongo, Bunkyo-ku, Tokyo 113-8421 Japan; 3 Division of Complex Biosystem Research, Department of Research and Development, Institute of Natural Medicine, University of Toyama, Toyama, Toyama 930-0194 Japan

**Keywords:** Skeletal muscle, R-spondin 3, Myokine, Type I fiber, Muscle regeneration

## Abstract

**Background:**

In recent years, skeletal muscle has been recognized not only as a locomotor organ but also as a secretory organ. The bioactive molecules it releases, known as myokines, act on distant organs through the circulation and locally on muscle tissue. Previously, we identified R-spondin 3 (Rspo3) as a Type I fiber-specific myokine that promotes myoblast differentiation into Type I fibers in vitro. In this study, we further investigated whether Rspo3 is involved in regulating Type I fiber determination in vivo.

**Methods and results:**

We generated tamoxifen-induced muscle-specific Rspo3-deficient mice and found that Rspo3 deficiency impaired Type I fiber determination during muscle regeneration. In contrast, transient overexpression of Rspo3 through in vivo electroporation enhanced the regeneration of Type I fibers, supporting its functional role in fiber-type specification.

**Conclusion:**

This work reveals that Rspo3 as a Type I fiber specific myokine promotes Type I fiber determination during muscle regeneration in vivo.

**Supplementary Information:**

The online version contains supplementary material available at 10.1007/s11033-025-11099-6.

## Introduction

Skeletal muscle, comprising approximately 40% of total body weight, plays a critical role in physical function and metabolic regulation [1]. Skeletal muscle exhibits a highly structured architecture, of long multinucleated structures known as muscle fibers. Muscle fibers are typically categorized into two major types—Type I (slow-twitch) and Type II (fast-twitch)—based on their distinct contractile dynamics and metabolic profiles [2]. Type I fibers contract slowly, resist fatigue, and primarily rely on oxidative metabolism [3]. In contrast, Type II fibers contract rapidly, fatigue quicker, and depend largely on glycolytic pathways. The contractile and metabolic properties of skeletal muscle are determined by fiber type distribution, which varies across different muscles [4]. For example, soleus muscle has a higher proportion of Type I fibers, whereas quadriceps muscle is rich in Type II fibers [5].

Satellite cells, the resident stem cells of adult muscle, are essential for preserving muscle mass during regeneration. Under normal conditions, satellite cells remain in a quiescent state; however, upon muscle injury, they become activated and undergo a myogenic program that ultimately leads to the formation of new fibers. The mechanisms by which immature myoblasts differentiate into myotubes and acquire distinct Type I or Type II fiber identities remain poorly understood. Nevertheless, certain transcription factors have been implicated in this process. For example, nuclear factor activated T cell 1 (NFATc1) promotes Type I fiber specification by activating fiber type–specific gene programs during regeneration [6], although the precise regulatory mechanisms driving this transcription factor remain unclear.

We recently discovered that Rspo3 is expressed exclusively in Type I fibers and secreted as a Type I–specific myokine. R-spondin 3 (Rspo3) acts on immature myoblasts to promote their differentiation into Type I fibers [7]. While these findings are intriguing, the observed induction of Type I fibers by Rspo3 was demonstrated only in vitro. Therefore, in the present study, we investigated whether the Type I fiber–inducing effect of Rspo3 observed in vitro is also recapitulated in vivo. Our results suggest that Rspo3 functions as a myokine mediating Type I fiber determination during muscle regeneration in adult muscle.

## Materials and methods

### Animal

Rspo3-floxed mice were purchased from the Jackson Laboratory (Rspo3tm1.1Jcob/J mice, Stock No: 027313; ME, USA). Muscle-specific Rspo3 knockout (Rspo3 mKO) mice were generated by crossing these with mice expressing Cre recombinase-estrogen receptor fusion protein (CreER) under the control of the HSA promoter (HSA-CreER mice). Littermates with floxed Rspo3 alleles but lacking the CreER transgene served as wild-type (WT) controls. Experimental cohorts were generated by sibling mating of HSA-CreER and WT genotype carriers. Mice were housed and bred in the animal facility of Tokyo Metropolitan University (Permit Numbers: A4-4, A5-9, A6-7, A7-018). All animals were maintained under SPF conditions. Animal handling and experimental procedures employed in this study strictly adhered to the guidelines of the Safety and Ethics Committee of Tokyo Metropolitan University. Research plans were executed after requisite approval. Plans for genetic recombinant experiments were sanctioned by the Genetic Recombinant Experiment Safety Committee of Tokyo Metropolitan University (Permit Numbers: G4-40, G5-5, G6-4, G7-21).

Experimental procedures used male mice. Rspo3 knockout was induced by administrating 10 µl/g body weight of tamoxifen (T5648; Sigma-Aldrich, MO, USA) solution at 10 mg/ml via intraperitoneal injection at 8-weeks-old for 5 consecutive days.

### Glucose tolerance test (GTT)

After overnight fasting, mice were weighed and rested for 1 h. A ~ 2 mm tail snip was used to measure fasting blood glucose with a One Touch Ultra glucometer (Life Scan, PA, USA). Mice then received an intraperitoneal glucose injection (100 mg/ml, 10 µl/g body weight). Blood glucose was measured at 15, 30, 60, 90, and 120 min post-injection, and results were plotted over time.

### Conventional PCR and real-time quantitative PCR

Total RNA was isolated from tissues using TRIzol reagent (Invitrogen, CA, USA). Complementary DNA (cDNA) was synthesized with PrimeScript™ First Strand cDNA Synthesis Kit (Takara, Shiga, Japan), as manufacturer’s protocol. Rspo3 mRNA expression was assessed by reverse transcription PCR (RT-PCR) using TaKaRa Taq polymerase (Takara) and gene-specific primer pairs. Rspo3 (Fw: GTACACTGTGAGGCCAGTGAA, Rv: ATGGCTAGAACACCTGTCCTG); GAPDH: (Fw: AACTTTGGCATTGTGGAAGG, Rv: ACACATTGGGGGTAGGAACA).

Quantitative real-time PCR (qRT-PCR) was conducted using CFX Opus 96 Real-Time PCR System (BIO-RAD, CA, USA) with DyNAmo ColorFlash SYBR Green qPCR Kit (Thermo Fisher Scientific). Primer sequences used are: Rspo3 (Fw: TGTCAGTATTGTACACTGTGAGGC, Rv: AGTTCTTGTCTCGCTGGTTGG); Lgr4 (Fw: AGAACTCAAAGTCCTAACCCTC, Rv: TCCTCCGGGACTGAGGTAAT); Lgr5 (Fw: GCCTTCAATCCCTGCGCCTA, Rv: CATGGCTTGCAGGGCTGATA); Lgr6 (Fw: GAGGACGGCATCATGCTGTC, Rv: GCTCCGTGAGGTTGTTCATACT); Tbp (Fw: AATGACTCCTATGACCCCTATCAC, Rv: AGGTCAAGTTTACAGCCAAGATTC).

### Immunoblotting

Tissue samples were lysed in buffer containing 50 mM Tris–HCl (pH 7.5), 1% NP-40, and additional reagents, and homogenized with a Polytron homogenizer (Kinematica, Lucerne, Switzerland). Homogenates were centrifuged at 13,000 × g for 15 min at 4 °C. The resulting supernatant was collected as tissue lysate, previously described [8]. Protein concentrations were quantified using Bradford assay (Bio-Rad, CA, USA). Equal amounts of protein were subjected to SDS-PAGE and transferred onto polyvinylidene difluoride (PVDF) membranes. Membranes were blocked with appropriate reagents followed by overnight incubation with primary antibodies at 4 °C. HRP-conjugated secondary antibodies (GE Healthcare, Buckinghamshire, UK) were applied for signal detection using enhanced chemiluminescence (ECL) reagents (PerkinElmer, MA, USA). Band intensities were quantified with ImageQuant TL software (Cytiva, MA, USA).

Antibodies used are: Myosin Heavy Chain (MyHC) I (M8421; Sigma, MO, USA), MyHC II (M4276; Sigma), PGC1α (sc-517380; SantaCruz, TX, USA), Hexokinase II (#2867; Cell Signaling Technology, MA, USA), CoxIV (#4850; Cell Signaling Technology), Myoglobin (ab77232; Abcam, Cambridge, UK), LGR4 (ab321789; Abcam), LGR5 (ab75850; Abcam), LGR6 (ab126747; Abcam), Rspo3 (17,193–1-AP; Proteintech, IL, USA), non-phosphorylated β-catenin (#8814; Cell Signaling Technology), GAPDH (#2118; Cell Signaling Technology), α-tubulin (#3873; Cell Signaling Technology).

### Immunofluorescence staining

Immunostaining followed our previous study [9]. Dried tissue sections were blocked in 5% goat serum (005–000–121; Fujifilm, Tokyo, Japan) in PBS at room temperature for 30 min, then incubated in a humidified chamber overnight at 4 °C with the following primary antibodies; MyHC I (M8421; Sigma). Samples were incubated for an hour with secondary antibodies Alexa Fluor 488 mouse IgG and Alexa Fluor 594 Rat IgG (Thermo Fisher Scientific, MA, USA). Sections were mounted with Vectashield Mounting Medium with DAPI (2-(4-amidinophenyl)−1H-indole-6-carboxamidine) (Vector Laboratories, CA, USA).

### Soleus injury induced by cardiotoxin injection

Following anesthesia, an incision was made in the skin and fascia of the posterior lower leg. Scissors were inserted into the space of the tendons and moved toward the knee to expose the soleus. Utilizing a microsyringe (Hamilton, NV, USA), 10 µl of 10 µM cardiotoxin (Funakoshi, Tokyo, Japan) was injected into the soleus of one leg along the long axis of the muscle fibers, the opposite leg underwent a sham procedure [10]. The incisions were adhered using biological adhesive (Konishi, Osaka, Japan). Mice were revived using Antisedan (Zenoaq, Fukushima, Japan), and housed with new bedding to mitigate infections.

### Cloning the R-spondin 3 gene

Mouse Rspo3 cDNA was cloned by RT-PCR-based method. The forward primer included the Kozac sequence (CACCATG) before the start codon of the coding sequence for mouse Rspo3 (Fw: GTCACCATGCACTTGCGACTGATTTC). The reverse primers included recognition sites to complement the coding sequence for the C-terminal end of mouse Rspo3 (Rv: GAGTCTACAGTAACCTCGCAGGA). Amplified fragments were cloned into the pCR2.1-TOPO vector (Thermo Fisher Scientific), digested with restriction enzymes EcoRI (Takara) and subcloned into the pCAGGS vector.

### DNA injection into regenerating Tibialis Anterior (TA) and in vivo electroporation

After anesthesia, 50 µl of 10 µM cardiotoxin was injected into the TA of 10-week-old WT mice to induce muscle injury. Three days later, 25 µl of 0.6 U/µl hyaluronidase (Sigma-Aldrich) in 0.9% saline was injected into the TA. [11]. After hyaluronidase injection, 25 µl of DNA plasmid (2 µg/µl in 0.9% saline) was injected into the TA along the muscle fiber axis. A stainless-steel needle electrode was then inserted, and eight 100 V pulses (20 ms, 1 Hz) were delivered using a pulse generator (Uchida Denshi, Hachioji, Japan). Mice were revived with Antisedan.

### Statistics

All data are shown as mean ± S.E.M (standard error of the mean). An unpaired Student’s t-test was performed to evaluate statistical differences between two groups. Values of p < 0.05 were considered to be statistically significant.

## Results

### Rspo3 ablation from adult mice did not affect whole-body metabolism or skeletal muscle composition

**Fig. 1:**
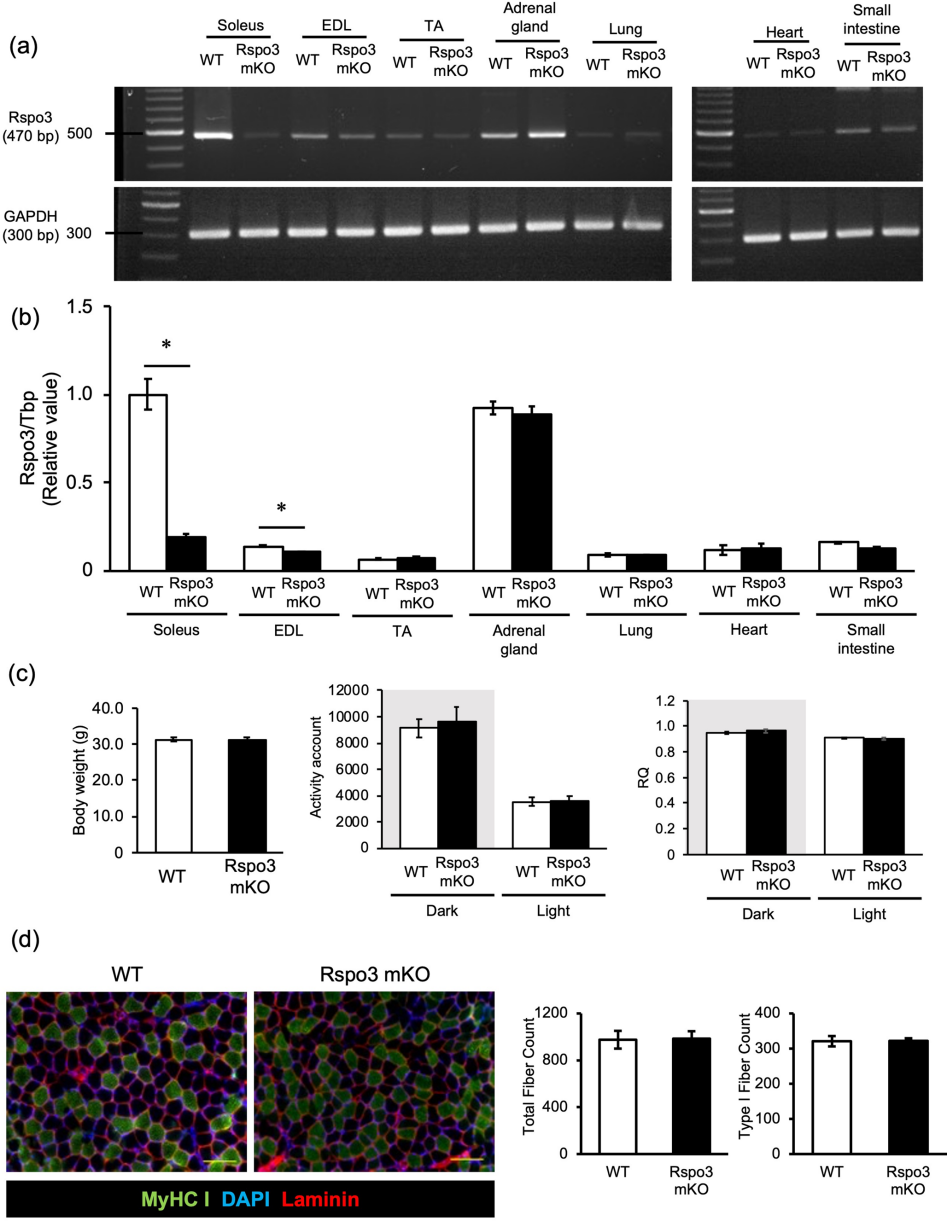
Whole-body metabolism and muscle composition were not altered in Rspo3 mKO mice compared to WT mice. **a** Rspo3 mRNA expression in muscles and other tissues from Rspo3 mKO and WT mice was detected by conventional RT-PCR and **b** quantified by quantitative RT-PCR. n = 4. **c** Body weight, activity account and RQ were measured. n = 4. **d** Representative immunostaining images for MyHC I (green), laminin (red) and nuclei (DAPI; blue) of soleus from Rspo3 mKO and WT mice, with total and Type I fiber counts found from the image. Scale bar is 100µm. n = 8–10. *; p < 0.05 by Student’s t-test, comparing Rspo3 mKO and WT mice. Values are presented as mean ± S.E.M

To assess Rspo3 gene ablation by tamoxifen, conventional and quantitative PCR were performed on skeletal muscle and other tissues from wild-type (WT) and muscle-specific Rspo3 knockout (mKO) mice two weeks post-treatment. Rspo3 expression was markedly reduced in the soleus, rich in Type I fibers, as shown by conventional (Fig. 1a) and quantitative PCR (Fig. 1b). In contrast, minimal changes were seen in Type II fiber–dominant muscles like TA and EDL, reflecting their low basal Rspo3 levels. Rspo3 expression in non-muscle tissues remained unaffected, confirming effective and muscle-specific ablation.

To characterize the metabolic phenotype of Rspo3 mKO mice, body weight, locomotor activity, and respiratory quotient (RQ: VCO₂/VO₂) were measured. No significant differences were observed between Rspo3 mKO and WT mice (Fig. 1c, Supplementary Fig. 1a). To assess whether Rspo3 ablation affects systemic glucose metabolism, a glucose tolerance test (GTT) was performed, which revealed no significant differences between Rspo3 mKO and WT mice (Supplementary Fig. 1b). These findings suggest that Rspo3 expression in muscle has limited impact on regulation of systemic metabolism.

To assess whether Rspo3 ablation affects fiber-type distribution, soleus muscles were collected from Rspo3 mKO and WT mice two weeks after tamoxifen treatment for fiber type composition analysis. The total number of muscle fibers and Type I fibers was similar between Rspo3 mKO and WT mice (Fig. 1d). Western blot confirmed comparable expression of MyHC I and II in soleus muscles (Supplementary Fig. 1c). Likewise, levels of oxidative metabolic proteins—PGC1α, HK II, COXIV, and Myoglobin—were unchanged in mKO mice. These results suggest Rspo3 is not involved in Type I fiber maintenance or phenotype transition in intact mature muscle.

### Rspo3 ablation impaired type I fiber determination during muscle regeneration

**Fig. 2:**
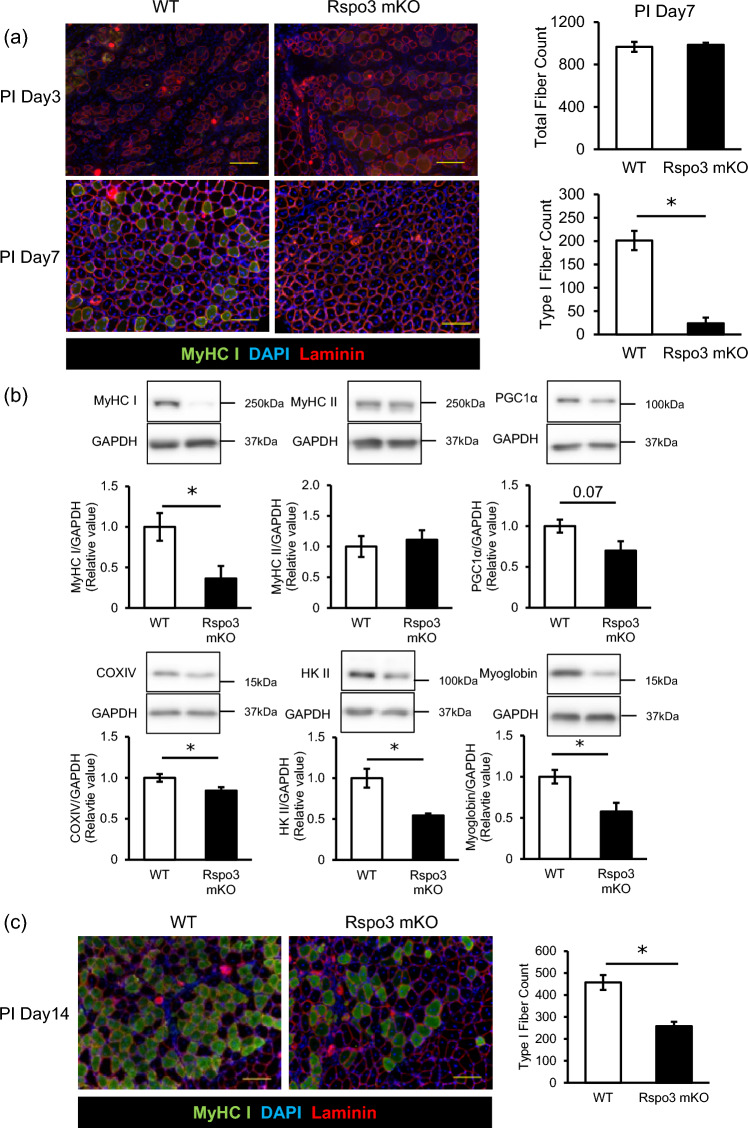
Rspo3 ablation impaired Type I fiber determination during muscle regeneration. **a** Representative immunostaining images for MyHC I (green), laminin (red) and nuclei (DAPI; blue) of soleus from Rspo3 mKO and WT mice on day 3 post-injury (PI Day 3) and day 7 (PI Day 7), with total and Type I fiber counts found from PI Day 7 immunostaining images. n = 3. **b** The expression levels of MyHC I, MyHC II, PGC1α, HK II, COX IV, and Myoglobin in soleus by PI Day 7 were measured by western blot. Protein expression levels were normalized to GAPDH. n = 3. **c** Representative immunostaining images of the soleus by day 14 post-injury (PI Day 14) from WT and Rspo3 mKO mice, with total and Type I fiber counts found from the images. Scale bar is 100µm. n = 8–10. *; p < 0.05 by Student’s t-test. Values are presented as mean ± S.E.M

We next investigated muscle regeneration, where satellite cells are activated, proliferate into myoblasts, and form new fibers. Since Rspo3 affects myoblasts but not differentiated myotubes in vitro [7], we hypothesized that Rspo3 may influence fiber-type distribution during regeneration. To test this, muscle injury was induced by cardiotoxin (CTX) injection into soleus muscle. Samples were examined on days 3, 7, and 14 post-injury. By day 3 post-injury, the basal lamina remained intact, but many fibers were necrotic with vacuolated cytoplasm, preventing fiber type identification (Fig. 2a). By day 7, newly formed centrally nucleated fibers emerged in the regenerating soleus (Fig. 2a). Type I fibers were present in WT mice, but were nearly absent in Rspo3 mKO mice, suggesting that Rspo3 ablation impaired Type I fiber determination during regeneration. Western blot analysis showed that MyHC I protein expression was lower in soleus from Rspo3 mKO mice than in WT mice, whereas MyHC II protein expression was comparable (Fig. 2b). Expression levels of PGC1α, HK II, COXIV, and myoglobin were reduced in the soleus from Rspo3 mKO mice compared to WT controls. Although by 14 days post-injury Type I fiber population in Rspo3 mKO mice began to recover, it remained less than that observed in WT mice (Fig. 2c). These results suggest that Rspo3 contributes to Type I fiber determination by regulating MyHC I expression. Additionally, Rspo3 directly or indirectly may contribute to oxidative metabolic protein expression.

### Rspo3 ablation did not alter the receptors LGR4/5/6 expression during muscle regeneration

**Fig. 3:**
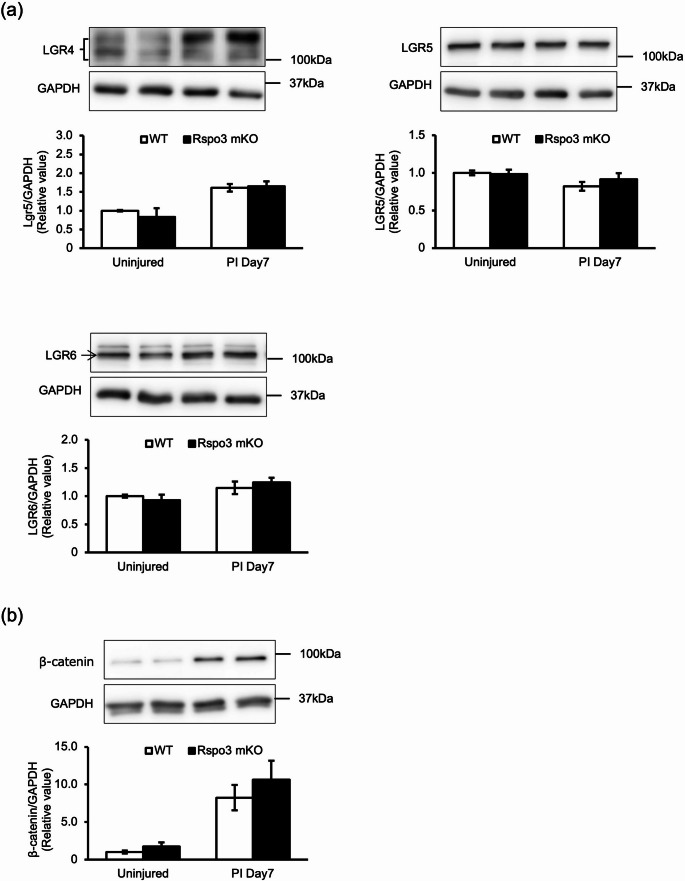
Rspo3 mKO and WT mice show comparable protein expression levels of Rspo3 receptors during muscle regeneration. **a** LGR4/5/6 expressions and **b** β-catenin expression in the soleus under uninjured conditions (Uninjured) and at day 7 post-injury (PI Day7) was measured by western blot. Expression levels were normalized to GAPDH; n = 3. Values are presented as mean ± S.E.M

Rspo3 acts by binding to receptors, including leucine-rich repeat-containing G-protein coupled receptor 4/5/6 (LGR4/5/6) [12]. To examine whether the reduction of Type I fibers in Rspo3 mKO mice results from insufficient receptor expression, we assessed LGR4/5/6 levels in both uninjured and regenerating soleus muscle. As a result, the protein and gene expressions of LGR4/5/6 were comparable between Rspo3 mKO and WT mice in uninjured and regenerating muscle (Fig. 3a and Supplementary Fig. 2a), indicating that impaired Type I fiber regeneration was due to the lack of Rspo3, rather than differences in receptor expression.

We next examined downstream signaling through LGR4/5/6. Rspo3 binding is known to potentiate Wnt signaling [13], leading to stabilization and accumulation of β-catenin. In intact muscle, β-catenin expression was low but increased rapidly after injury (Fig. 3b). However, no significant differences were observed between Rspo3 mKO and WT mice.

### Overexpressing Rspo3 in regenerating TA enhanced type I fiber determination

**Fig. 4:**
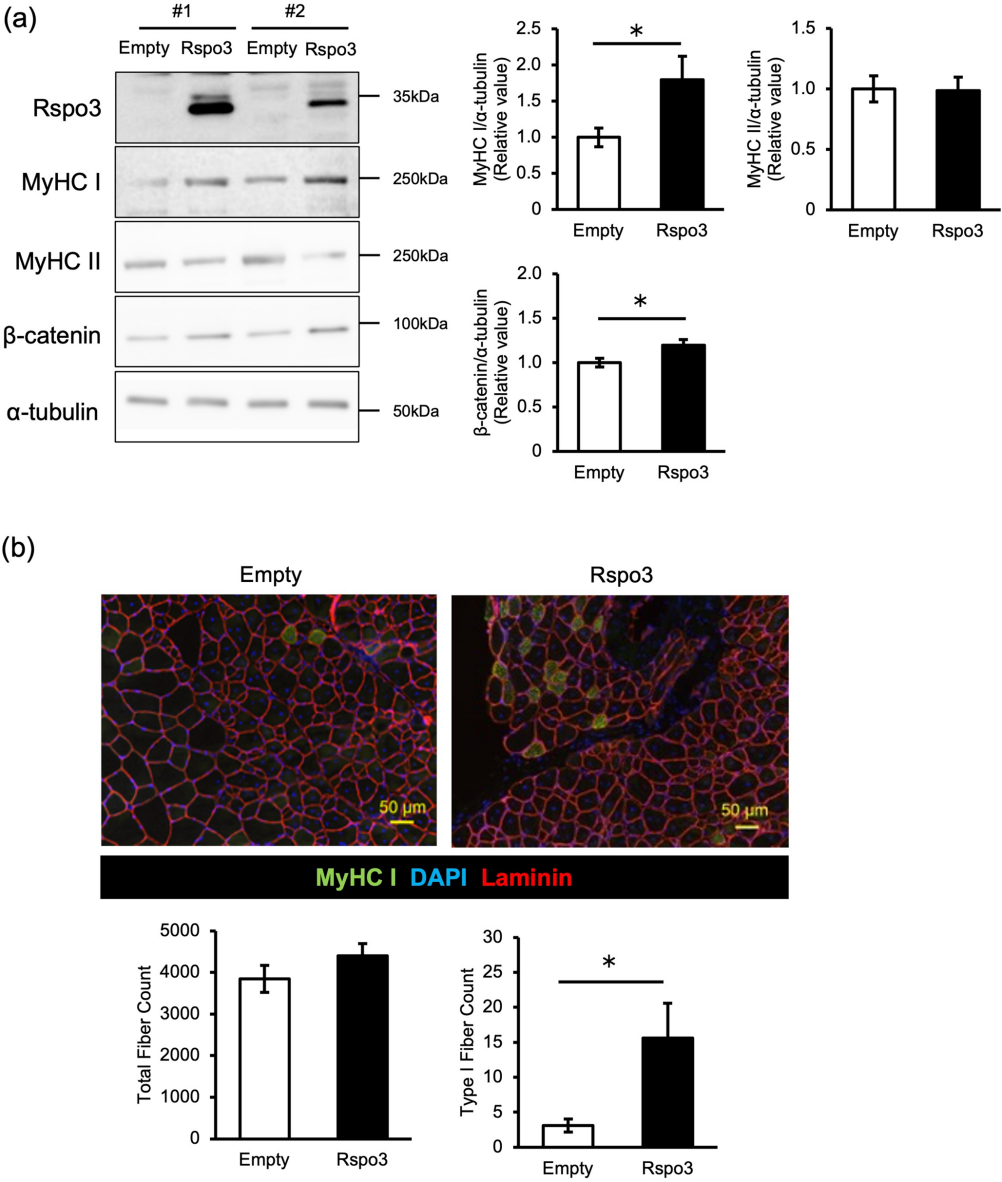
Overexpressing Rspo3 in regenerating TA enhanced Type I fiber determination. **a** Expression of Rspo3, MyHC I, MyHC II and β-catenin in regenerated TA was measured by western blot. Expression levels were normalized to α-tubulin. #1 and #2 stand for individual mouse #1 and mouse #2 **b** Representative immunostaining images for MyHC I (green), laminin (red) and nuclei (DAPI; blue) of regenerated TA overexpressing Rspo3 and controls, with calculated Type I fiber count. Scale bar is 50 µm. n = 10. *; p < 0.05 by Student’s t-test. Values are presented as mean ± S.E.M

We next tested whether Rspo3 overexpression promotes Type I fiber formation during regeneration. Rspo3 was electroporated into TA muscles three days post-injury, and muscles were analyzed 14 days later. Western blot confirmed robust Rspo3 protein overexpression in Rspo3-vector-injected muscle compared to empty-vector-injected muscle (Fig. 4a), accompanied by significant increase in MyHC I and β-catenin expression. Immunostaining of muscle cross-sections further revealed a marked increase of Type I fiber population in Rspo3-overexpressing muscles compared to controls (Fig. 4b). These results support the role of Rspo3 in promoting Type I fiber determination during muscle regeneration.

## Discussion

The present study investigated the physiological significance of Rspo3 using gain- and loss-of-function approaches in vivo. In brief Rspo3, as a Type I fiber specific myokine, mediates Type I fiber determination during muscle regeneration.

Rspo3 deficiency did not alter adult muscle fiber composition in vivo, which is consistent with our previous findings showing that Rspo3 affects myoblasts but not differentiated myotubes in vitro [7]. In contrast, Rspo3 deficiency impaired the Type I fiber regeneration. Following injury, satellite cells are activated, proliferate, and differentiate into muscle fibers with distinct properties. In this context, the absence of Rspo3 compromises the induction of Type I fibers. Notably, we previously found that Brain-Derived Neurotrophic Factor (BDNF) also act temporally during myogenesis: it does not alter myoblast behavior but instead inhibits differentiated myotube from committing to Type II fibers [14]. All these findings suggest many myokines may act in a phase-specific manner during muscle development.

One possible explanation for the regeneration defect in Rspo3 mKO mice is altered receptor expression. However, LGR4/5/6 protein expression levels were comparable between Rspo3 mKO and WT mice during regeneration, indicating that the impaired regeneration of Type I fiber is not due to receptor expression, but rather to altered induction of downstream molecules resulting from Rspo3 deficiency. To further address this, we examined the expression of β-catenin, the downstream effector of LGR4/5/6. β-catenin expression was rapidly upregulated following injury, consistent with a previous report of transient Wnt/β-catenin activation in regenerating myoblasts [15]. However, because regenerating soleus from Rspo3 mKO and WT mice expressed a comparable amount of β-catenin, it is unlikely that the expression level alone accounts for the downstream effects of Rspo3 deficiency. This finding underscores the complexity of the in vivo muscle environment and the difficulty of isolating signaling deficits specifically attributable to Rspo3 deficiency. Notably, β-catenin expression was minimal in uninjured muscle, indicating a limited role in muscle homeostasis. This may explain why Rspo3 deficiency in uninjured muscle did not alter fiber type, as its downstream signaling remains inactive under basal conditions.

At 14 days post-injury, Rspo3 mKO mice exhibited partial recovery of the Type I muscle fibers, suggesting other factors, besides Rspo3, also regulate Type I fiber determination during regeneration. For instance, peroxisome-proliferator-activated receptor-γ co-activator-1 (PGC-1α), with myocyte enhancer factor-2 (MEF2) and estrogen-related receptor alpha (ERRα), promotes Type I fiber determination by enhancing mitochondrial biogenesis and oxidative metabolism [16, 17]. Taken together, these findings highlight that fiber-type regulation involves the integration of multiple signaling pathways that converge within skeletal muscle to orchestrate fiber-type determination.

## Conclusion

This work provides new insight into the molecular mechanisms governing fiber-type plasticity and highlights Rspo3 as a Type I fiber specific myokine that mediates Type I fiber determination during muscle regeneration.

## Supplementary Information

Below is the link to the electronic supplementary material.

Supplementary file1 (PPTX 4172 KB). Supplementary Fig. 1. Energy expenditure and systemic glucose metabolism were not altered in Rspo3 mKO mice compared to WT mice. (a) Carbohydrate (CHO) consumption, fat consumption and energy expenditure were measured. (b) Blood glucose concentration after glucose solution injection was plotted. n = 4. (c) The expression levels of MyHC I, MyHC II, PGC1α, HK II, COX IV and Myoglobin in the soleus after Rspo3 ablation were measured by western blot. Protein expression levels were normalized to GAPDH. n = 8-10. Values are presented as mean ± S.E.M. Supplementary Fig. 2. Rspo3 mKO and WT mice show comparable LGR4/5/6 mRNA expression levels during muscle regeneration. (a) LGR4, LGR5 and LGR6 mRNA expressions in soleus under uninjured conditions (Uninjured) and post-injury day 7 (PI Day 7) were quantified by quantitative RT-PCR. n = 3. Values are presented as mean ± S.E.M

Supplementary file2 (PPTX 53752 KB)

## Data Availability

No datasets were generated or analyzed during the current study. All data generated or analyzed during this study are included in this published article.

## References

[1] Argilés JM et al (2016) Skeletal muscle regulates metabolism via interorgan crosstalk: roles in health and disease. J Am Med Dir Assoc 17(9):789–796. 10.1016/j.jamda.2016.04.019 27324808 10.1016/j.jamda.2016.04.019

[2] Schiaffino S, Reggiani C (2011) Fiber types in mammalian skeletal muscles. Physiol Rev 91(4):1447–1531. 10.1152/physrev.00031.2010 22013216 10.1152/physrev.00031.2010

[3] Harridge SDR et al (1996) Whole-muscle and single-fibre contractile properties and myosin heavy chain isoforms in humans. Pflugers Arch 432:913–920. 10.1007/s004240050215 8772143 10.1007/s004240050215

[4] Bottinelli RYCR, Reggiani C (2000) Human skeletal muscle fibres: molecular and functional diversity. Prog Biophys Mol Biol 73(2–4):195–262. 10.1016/s0079-6107(00)00006-7 10958931 10.1016/s0079-6107(00)00006-7

[5] Hettige Pabodha et al (2020) Comparative analysis of the transcriptomes of EDL, psoas, and soleus muscles from mice. BMC Genom 21:1–16. 10.1186/s12864-020-07225-2 10.1186/s12864-020-07225-2 PMC7678079 33213377

[6] Shin J et al (2023) Specification of skeletal muscle fiber-type is determined by the calcineurin/NFATc1 signaling pathway during muscle regeneration. Biochem Biophys Res Commun 659:20–28. 10.1016/j.bbrc.2023.03.032 37031590 10.1016/j.bbrc.2023.03.032

[7] Mita Y et al (2022) R-spondin3 is a myokine that differentiates myoblasts to type I fibres. Sci Rep 12(1):13020. 10.1038/s41598-022-16640-2 35906363 10.1038/s41598-022-16640-2 PMC9338073

[8] Manabe Y et al (2014) Redox proteins are constitutively secreted by skeletal muscle. J Physiol Sci 64(6):401–409. 10.1007/s12576-014-0334-7 25205643 10.1007/s12576-014-0334-7 PMC10717412

[9] Sawano S et al (2016) A one-step immunostaining method to visualize rodent muscle fiber type within a single specimen. PLoS ONE 11(11):e0166080. 10.1371/journal.pone.0166080 27814384 10.1371/journal.pone.0166080 PMC5096669

[10] Fletcher JE et al (1991) Effects of a cardiotoxin from *Naja naja kaouthia* venom on skeletal muscle: involvement of calcium-induced calcium release, sodium ion currents and phospholipases A2 and C. Toxicon 29(12):1489–1500. 10.1016/0041-0101(91)90005-c 1666202 10.1016/0041-0101(91)90005-c

[11] Akerstrom T et al (2015) Optimizing hyaluronidase dose and plasmid DNA delivery greatly improves gene electrotransfer efficiency in rat skeletal muscle. Biochem Biophys Rep 4:342–350. 10.1016/j.bbrep.2015.10.007 29124223 10.1016/j.bbrep.2015.10.007 PMC5669402

[12] Wang D et al (2013) Structural basis for R-spondin recognition by LGR4/5/6 receptors. Genes Dev 27(12):1339–1344. 10.1101/gad.219360.113 23756652 10.1101/gad.219360.113 PMC3701189

[13] Mah AT, Yan KS, Kuo CJ (2016) Wnt pathway regulation of intestinal stem cells. J Physiol 594(17):4837–4847. 10.1113/JP271754 27581568 10.1113/JP271754 PMC5009769

[14] Teng Hu et al (2024) Myokine BDNF highly expressed in type I fibers inhibits the differentiation of myotubes into type II fibers. Mol Biol Rep 51(1):1143. 10.1007/s11033-024-10044-3 39531063 10.1007/s11033-024-10044-3 PMC11557626

[15] Murphy MM et al (2014) Transiently active Wnt/β-catenin signaling is not required but must be silenced for stem cell function during muscle regeneration. Stem Cell Reports 3(3):475–488. 10.1016/j.stemcr.2014.06.019 25241745 10.1016/j.stemcr.2014.06.019 PMC4266007

[16] Lin Jiandie et al (2002) Transcriptional co-activator PGC-1α drives the formation of slow-twitch muscle fibres. Nature 418(6899):797–801. 10.1007/s00424-007-0206-6 12181572 10.1038/nature00904

[17] Czubryt MP et al (2003) Regulation of peroxisome proliferator-activated receptor γ coactivator 1α (PGC-1α) and mitochondrial function by MEF2 and HDAC5. Proc Natl Acad Sci U S A 100(4):1711–1716. 10.1073/pnas.0337639100 12578979 10.1073/pnas.0337639100 PMC149898

