# Supplementary material for: R-spondin 3 is a myokine that mediates type I fiber determination during skeletal muscle regeneration
Source: Mol Biol Rep. 2025 Oct 9;52(1):995. doi: 10.1007/s11033-025-11099-6 (PMC12511182; doi:10.1007/s11033-025-11099-6)
Supplement: Supplementary file 1 — Supplementary file1 (PPTX 4172 KB). Supplementary Fig. 1. Energy expenditure and systemic glucose metabolism were not altered in Rspo3 mKO mice compared to WT mice. (a) Carbohydrate (CHO) consumption, fat consumption and energy expenditure were measured. (b) Blood glucose concentration after glucose solution injection was plotted. n = 4. (c) The expression levels of MyHC I, MyHC II, PGC1α, HK II, COX IV and Myoglobin in the soleus after Rspo3 ablation were measured by western blot. Protein expression levels were normalized to GAPDH. n = 8-10. Values are presented as mean ± S.E.M. Supplementary Fig. 2. Rspo3 mKO and WT mice show comparable LGR4/5/6 mRNA expression levels during muscle regeneration. (a) LGR4, LGR5 and LGR6 mRNA expressions in soleus under uninjured conditions (Uninjured) and post-injury day 7 (PI Day 7) were quantified by quantitative RT-PCR. n = 3. Values are presented as mean ± S.E.M [file 11033_2025_11099_MOESM1_ESM.pptx]

## Slide 1
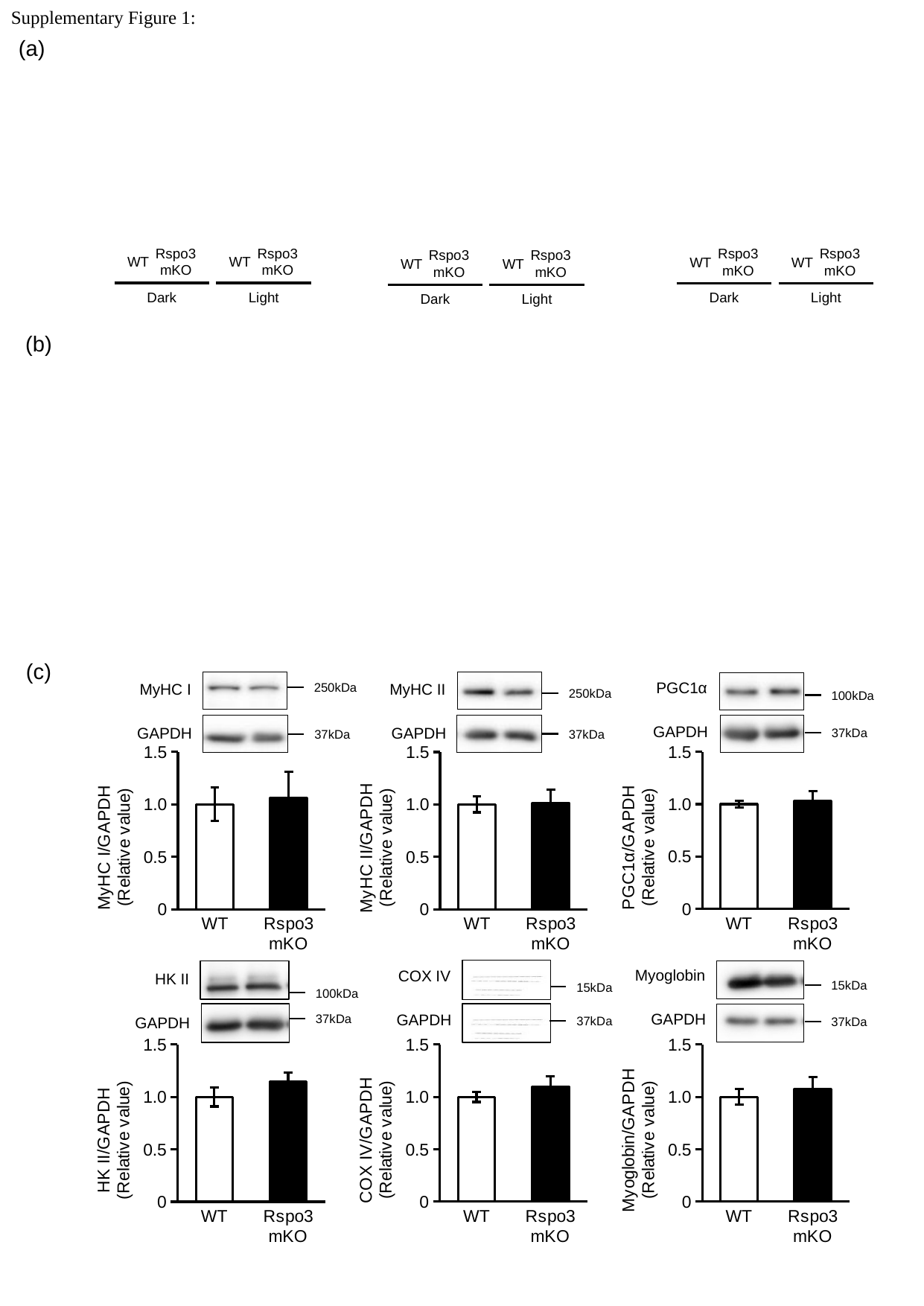

Supplementary Figure 1:
(a)
Rspo3
mKO
WT
Dark
Rspo3
mKO
WT
Light
Rspo3
mKO
WT
Dark
Rspo3
mKO
WT
Light
Rspo3
mKO
WT
Dark
Rspo3
mKO
WT
Light
(b)
(c)
PGC1α
MyHC II
MyHC I
250kDa
250kDa
100kDa
GAPDH
GAPDH
GAPDH
37kDa
37kDa
37kDa
### Chart
| Category | |
|---|---|
| WT | 1.0 |
| Rspo3 mKO | 1.063103746148922 |
### Chart
| Category | |
|---|---|
| WT | 1.0 |
| Rspo3 mKO | 1.0298665703790542 |
### Chart
| Category | |
|---|---|
| WT | 1.0 |
| Rspo3 mKO | 1.0102365530908706 |Myoglobin
COX IV
HK II
15kDa
15kDa
100kDa
GAPDH
GAPDH
37kDa
GAPDH
37kDa
37kDa
### Chart
| Category | |
|---|---|
| WT | 1.0 |
| Rspo3 mKO | 1.148609659249613 |
### Chart
| Category | |
|---|---|
| WT | 1.0 |
| Rspo3 mKO | 1.0985719659320916 |
### Chart
| Category | |
|---|---|
| WT | 0.9999999999999997 |
| Rspo3 mKO | 1.0723047725517454 |

## Slide 2
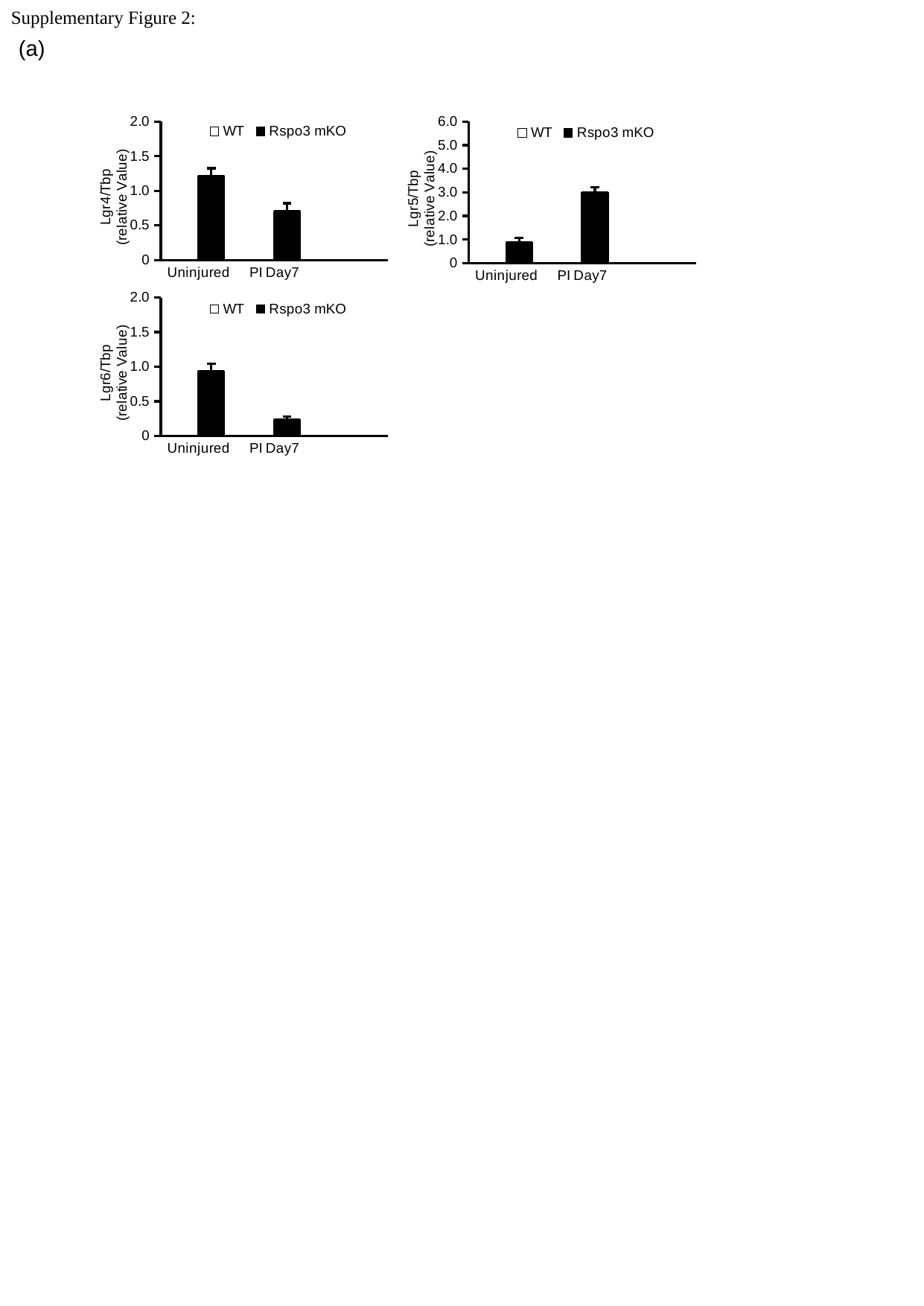

Supplementary Figure 2:
(a)
### Chart
| Category | | |
|---|---|---|
| Uninjured | 1.0000000000000002 | 1.2072585616690643 |
| PI Day7 | 0.6927624616642184 | 0.7020349441259338 |
### Chart
| Category | | |
|---|---|---|
| Uninjured | 1.0 | 0.8935801584678401 |
| PI Day7 | 3.671551989168059 | 2.991235821835058 |
### Chart
| Category | | |
|---|---|---|
| Uninjured | 1.0 | 0.9369389216154996 |
| PI Day7 | 0.18290099104104654 | 0.2312913535146912 |
