# Supplementary material for: R-spondin 3 is a myokine that mediates type I fiber determination during skeletal muscle regeneration
Source: Mol Biol Rep. 2025 Oct 9;52(1):995. doi: 10.1007/s11033-025-11099-6 (PMC12511182; doi:10.1007/s11033-025-11099-6)
Supplement: Supplementary file 2 — Supplementary file2 (PPTX 53752 KB) [file 11033_2025_11099_MOESM2_ESM.pptx]

## Slide 1
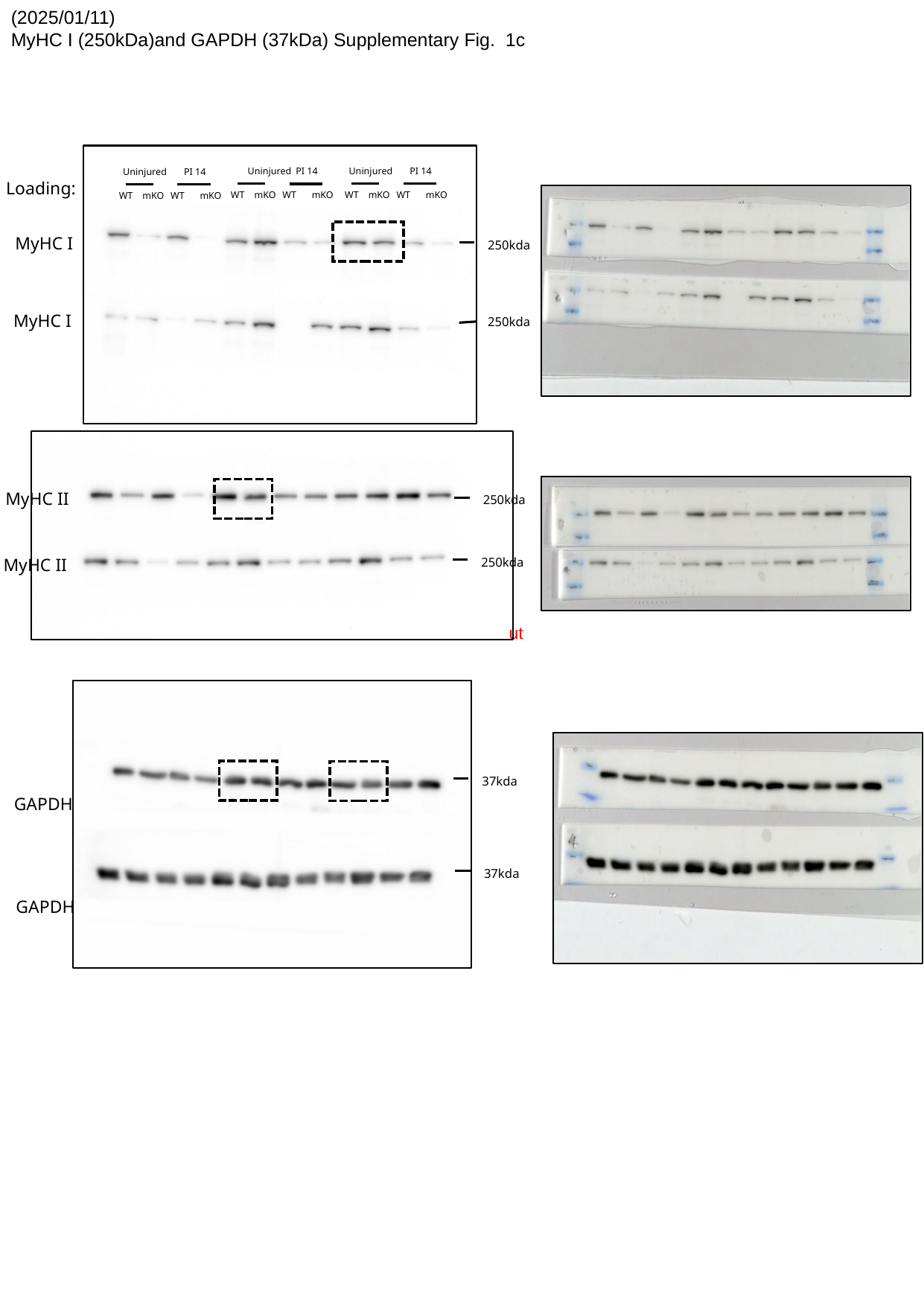

(2025/01/11)
MyHC I (250kDa)and GAPDH (37kDa) Supplementary Fig. 1c
Uninjured
WT
mKO
PI 14
mKO
WT
Uninjured
WT
mKO
PI 14
mKO
WT
Uninjured
WT
mKO
PI 14
mKO
WT
Loading:
MyHC I
250kda
MyHC I
250kda
MyHC II
250kda
MyHC II
250kda
Cut
37kda
GAPDH
37kda
GAPDH

## Slide 2
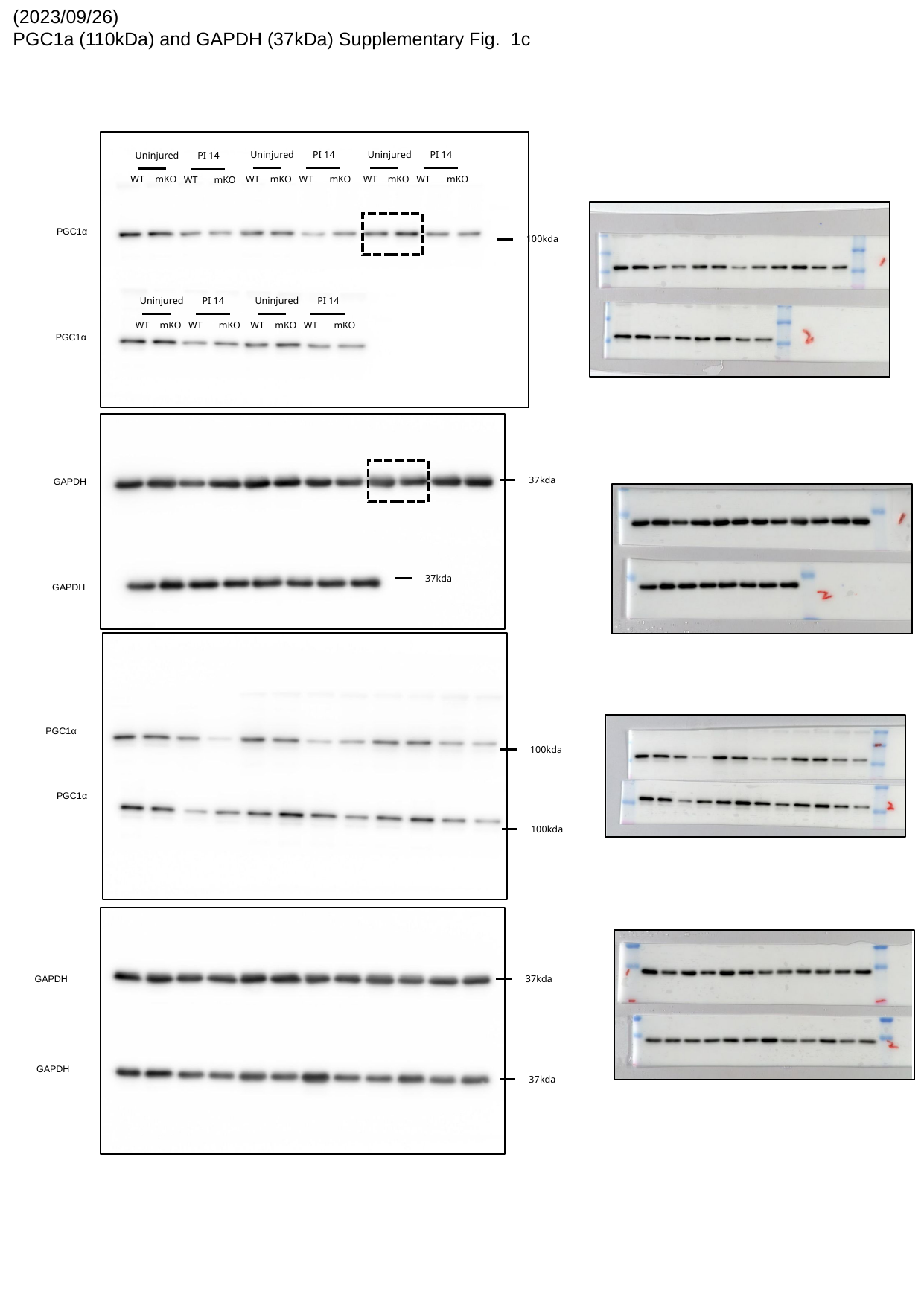

(2023/09/26)
PGC1a (110kDa) and GAPDH (37kDa) Supplementary Fig. 1c
Uninjured
WT
mKO
PI 14
mKO
WT
Uninjured
WT
mKO
PI 14
mKO
WT
Uninjured
WT
mKO
PI 14
mKO
WT
PGC1α
100kda
Uninjured
WT
mKO
PI 14
mKO
WT
Uninjured
WT
mKO
PI 14
mKO
WT
PGC1α
37kda
GAPDH
37kda
GAPDH
PGC1α
100kda
PGC1α
100kda
GAPDH
37kda
GAPDH
37kda

## Slide 3
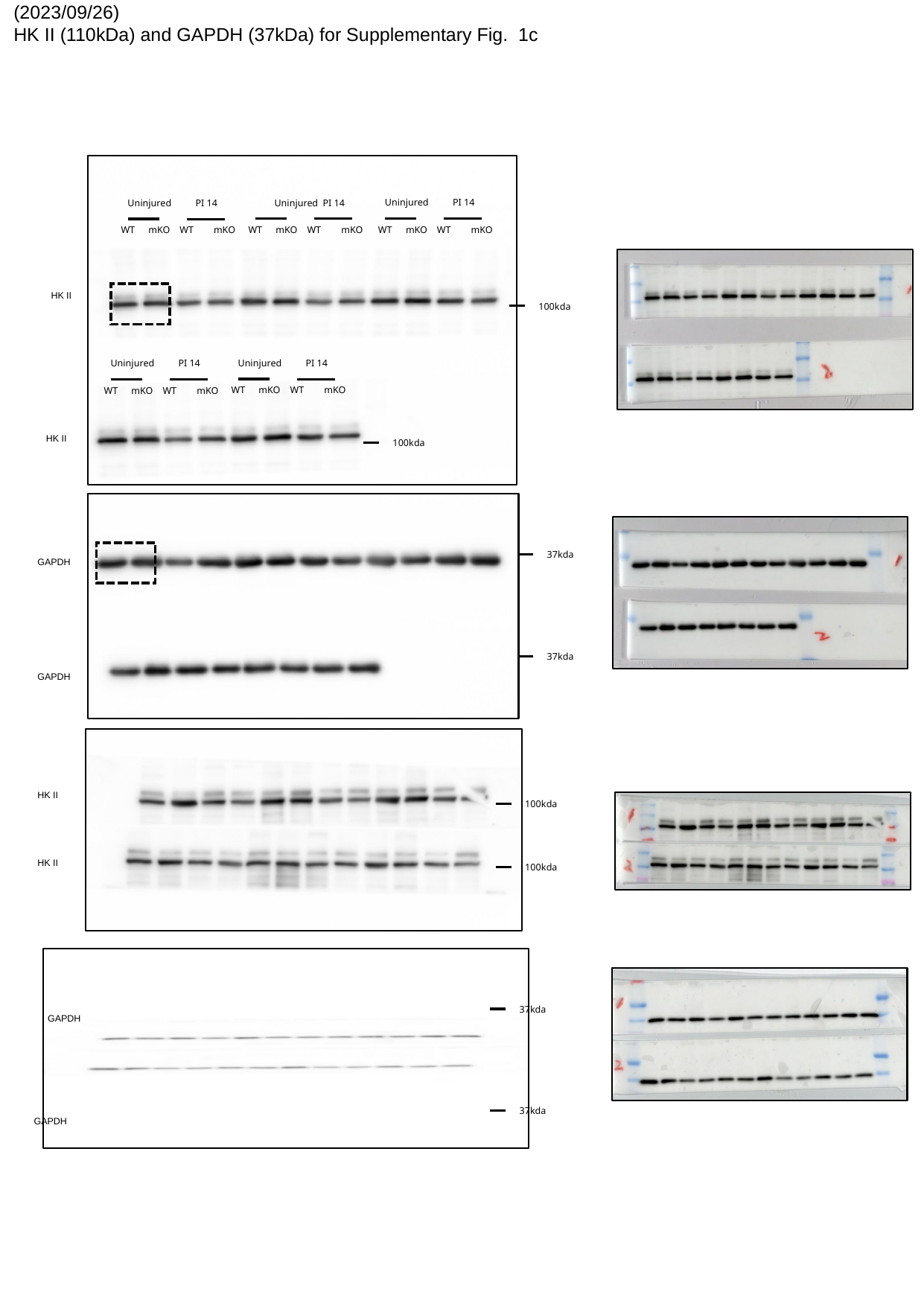

(2023/09/26)
HK II (110kDa) and GAPDH (37kDa) for Supplementary Fig. 1c
Uninjured
WT
mKO
PI 14
mKO
WT
Uninjured
WT
mKO
PI 14
mKO
WT
Uninjured
WT
mKO
PI 14
mKO
WT
HK II
100kda
Uninjured
WT
mKO
PI 14
mKO
WT
Uninjured
WT
mKO
PI 14
mKO
WT
HK II
100kda
37kda
GAPDH
37kda
GAPDH
HK II
100kda
HK II
100kda
37kda
GAPDH
37kda
GAPDH

## Slide 4
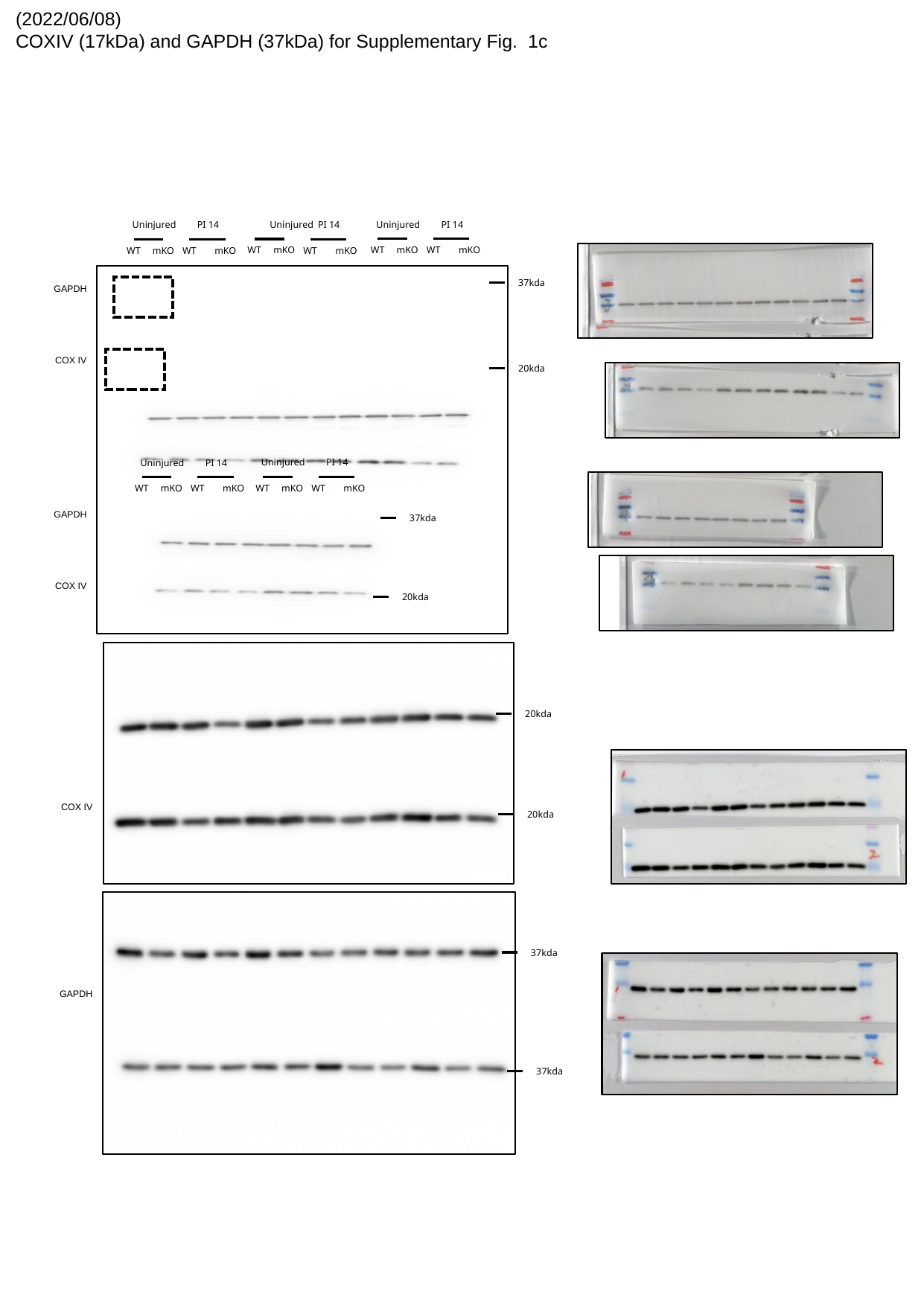

(2022/06/08)
COXIV (17kDa) and GAPDH (37kDa) for Supplementary Fig. 1c
Uninjured
WT
mKO
PI 14
mKO
WT
Uninjured
WT
mKO
PI 14
mKO
WT
Uninjured
WT
mKO
PI 14
mKO
WT
37kda
GAPDH
COX IV
20kda
Uninjured
WT
mKO
PI 14
mKO
WT
Uninjured
WT
mKO
PI 14
mKO
WT
GAPDH
37kda
COX IV
20kda
20kda
COX IV
20kda
37kda
GAPDH
37kda

## Slide 5
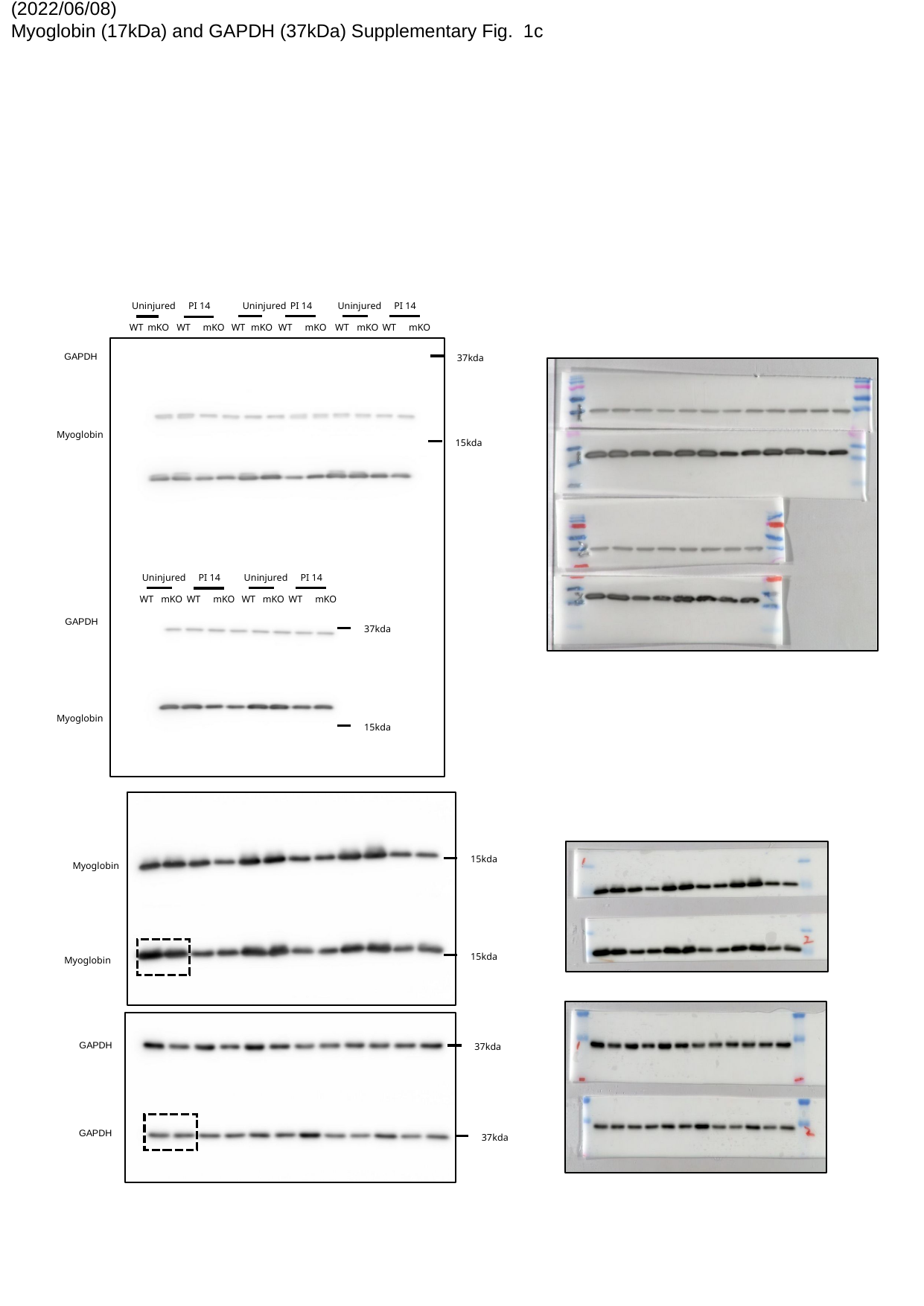

(2022/06/08)
Myoglobin (17kDa) and GAPDH (37kDa) Supplementary Fig. 1c
Uninjured
WT
mKO
PI 14
mKO
WT
Uninjured
WT
mKO
PI 14
mKO
WT
Uninjured
WT
mKO
PI 14
mKO
WT
GAPDH
37kda
Myoglobin
15kda
Uninjured
WT
mKO
PI 14
mKO
WT
Uninjured
WT
mKO
PI 14
mKO
WT
GAPDH
37kda
Myoglobin
15kda
15kda
Myoglobin
15kda
Myoglobin
GAPDH
37kda
GAPDH
37kda

## Slide 6
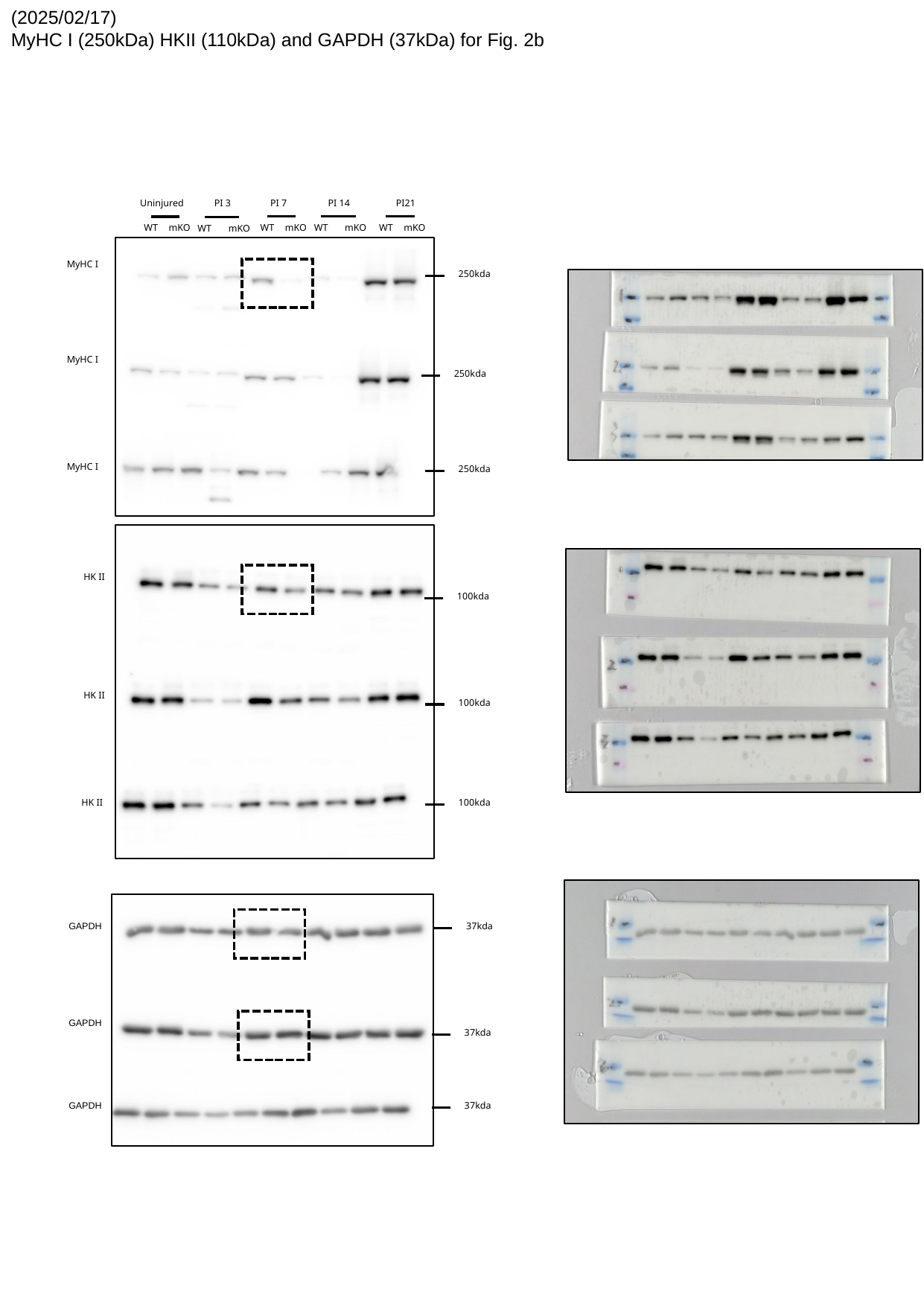

(2025/02/17)
MyHC I (250kDa) HKII (110kDa) and GAPDH (37kDa) for Fig. 2b
PI21
WT
mKO
PI 14
mKO
WT
PI 7
WT
mKO
Uninjured
WT
mKO
PI 3
mKO
WT
MyHC I
250kda
MyHC I
250kda
MyHC I
250kda
HK II
100kda
HK II
100kda
HK II
100kda
37kda
GAPDH
GAPDH
37kda
GAPDH
37kda

## Slide 7
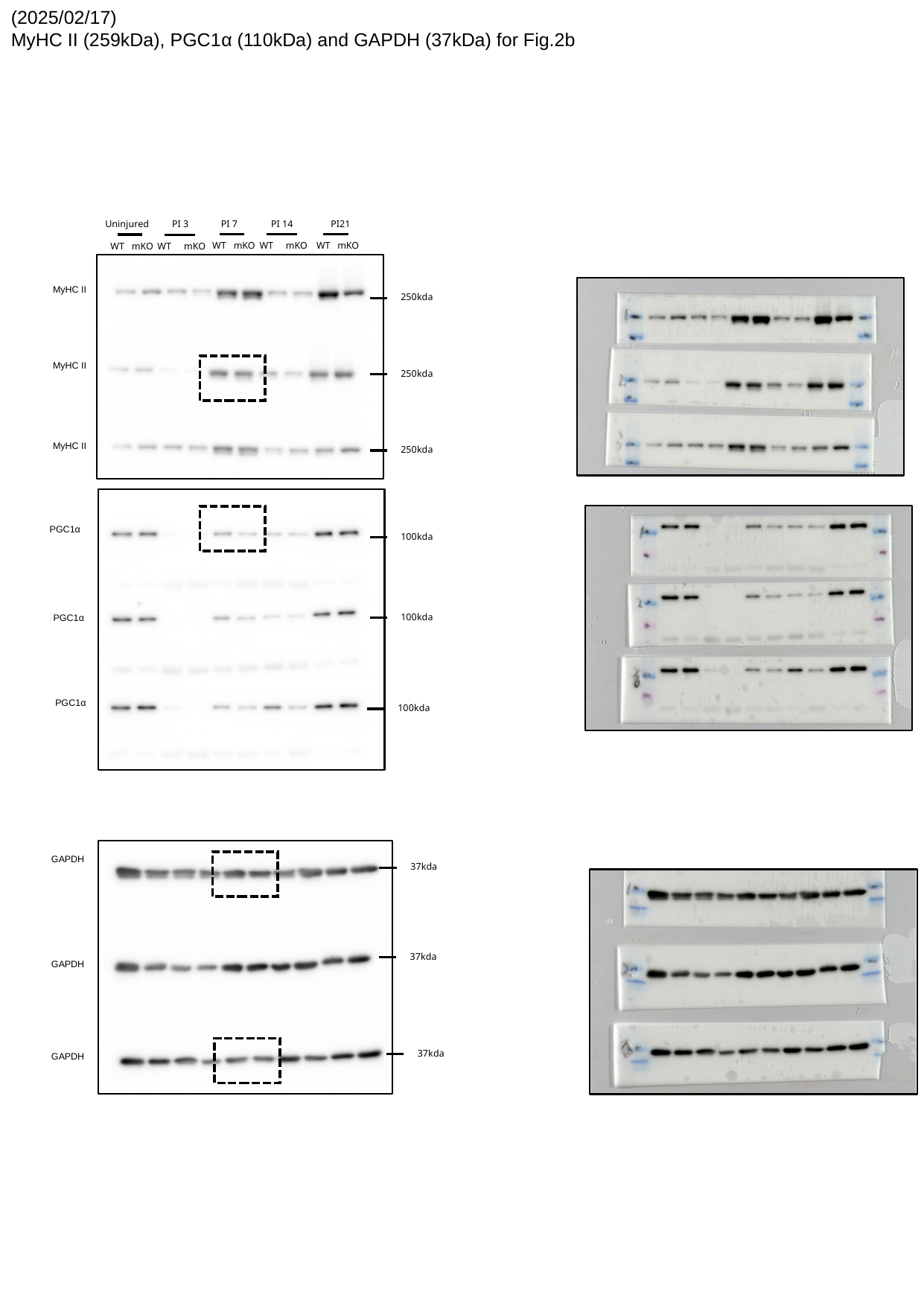

(2025/02/17)
MyHC II (259kDa), PGC1α (110kDa) and GAPDH (37kDa) for Fig.2b
PI21
mKO
WT
PI 14
mKO
WT
PI 7
WT
mKO
Uninjured
WT
mKO
PI 3
mKO
WT
MyHC II
250kda
MyHC II
250kda
MyHC II
250kda
PGC1α
100kda
100kda
PGC1α
PGC1α
100kda
GAPDH
37kda
37kda
GAPDH
37kda
GAPDH

## Slide 8
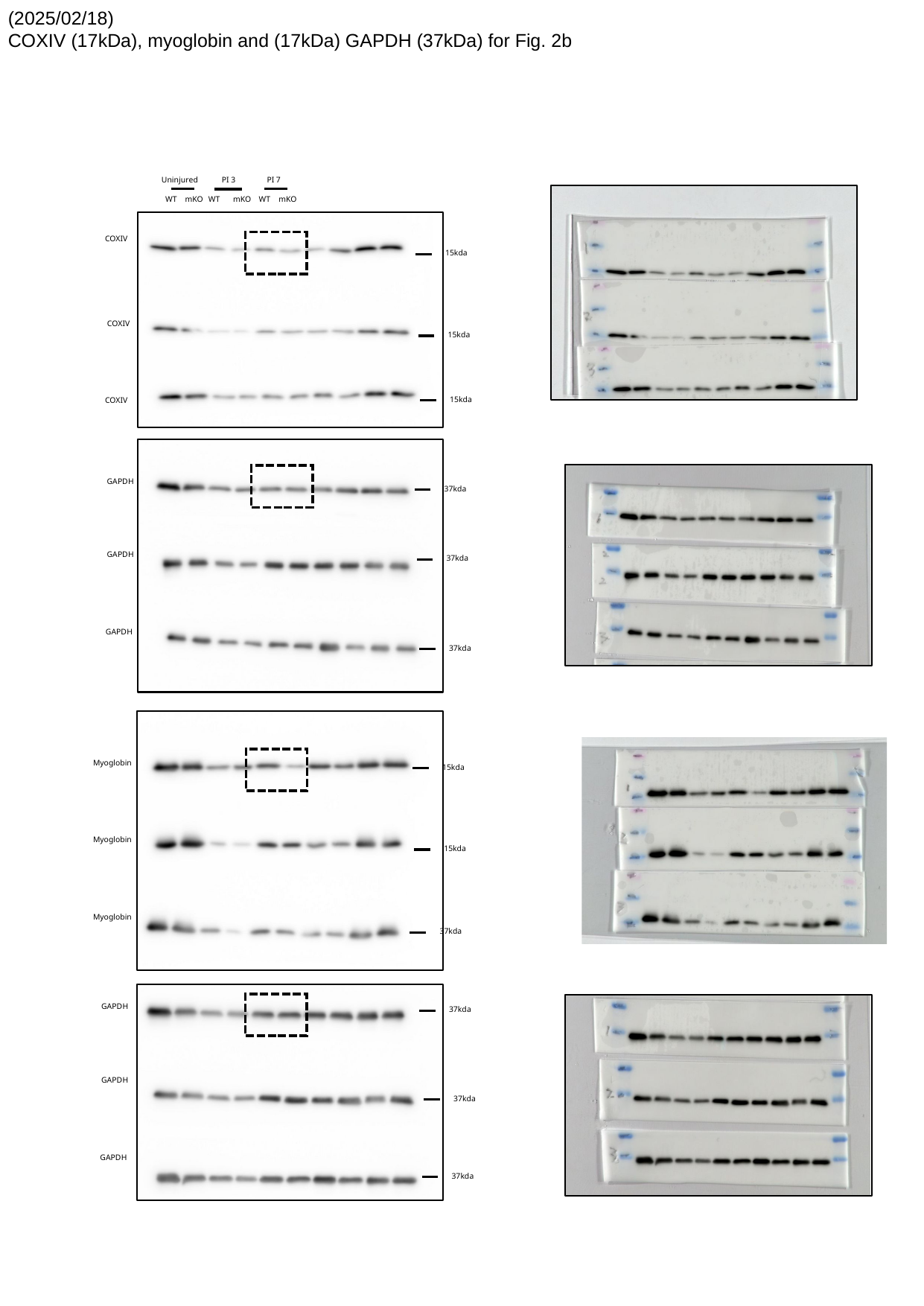

(2025/02/18)
COXIV (17kDa), myoglobin and (17kDa) GAPDH (37kDa) for Fig. 2b
Uninjured
mKO
WT
PI 3
WT
mKO
PI 7
WT
mKO
COXIV
15kda
COXIV
15kda
15kda
COXIV
GAPDH
37kda
GAPDH
37kda
GAPDH
37kda
Myoglobin
15kda
Myoglobin
15kda
Myoglobin
37kda
GAPDH
37kda
GAPDH
37kda
GAPDH
37kda

## Slide 9
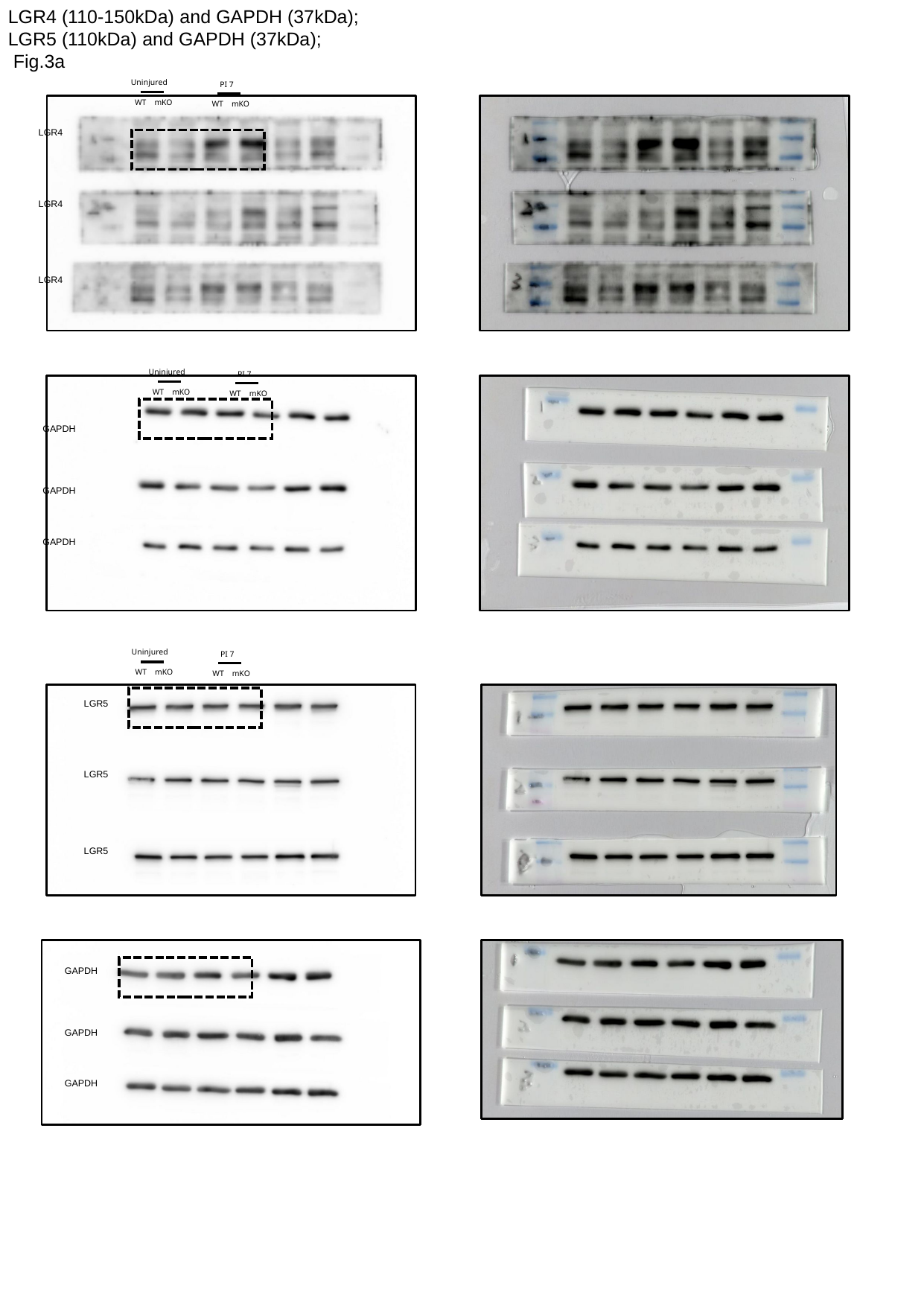

LGR4 (110-150kDa) and GAPDH (37kDa);
LGR5 (110kDa) and GAPDH (37kDa);
 Fig.3a
Uninjured
mKO
WT
PI 7
WT
mKO
LGR4
LGR4
LGR4
Uninjured
mKO
WT
PI 7
WT
mKO
GAPDH
GAPDH
GAPDH
Uninjured
mKO
WT
PI 7
WT
mKO
LGR5
LGR5
LGR5
GAPDH
GAPDH
GAPDH

## Slide 10
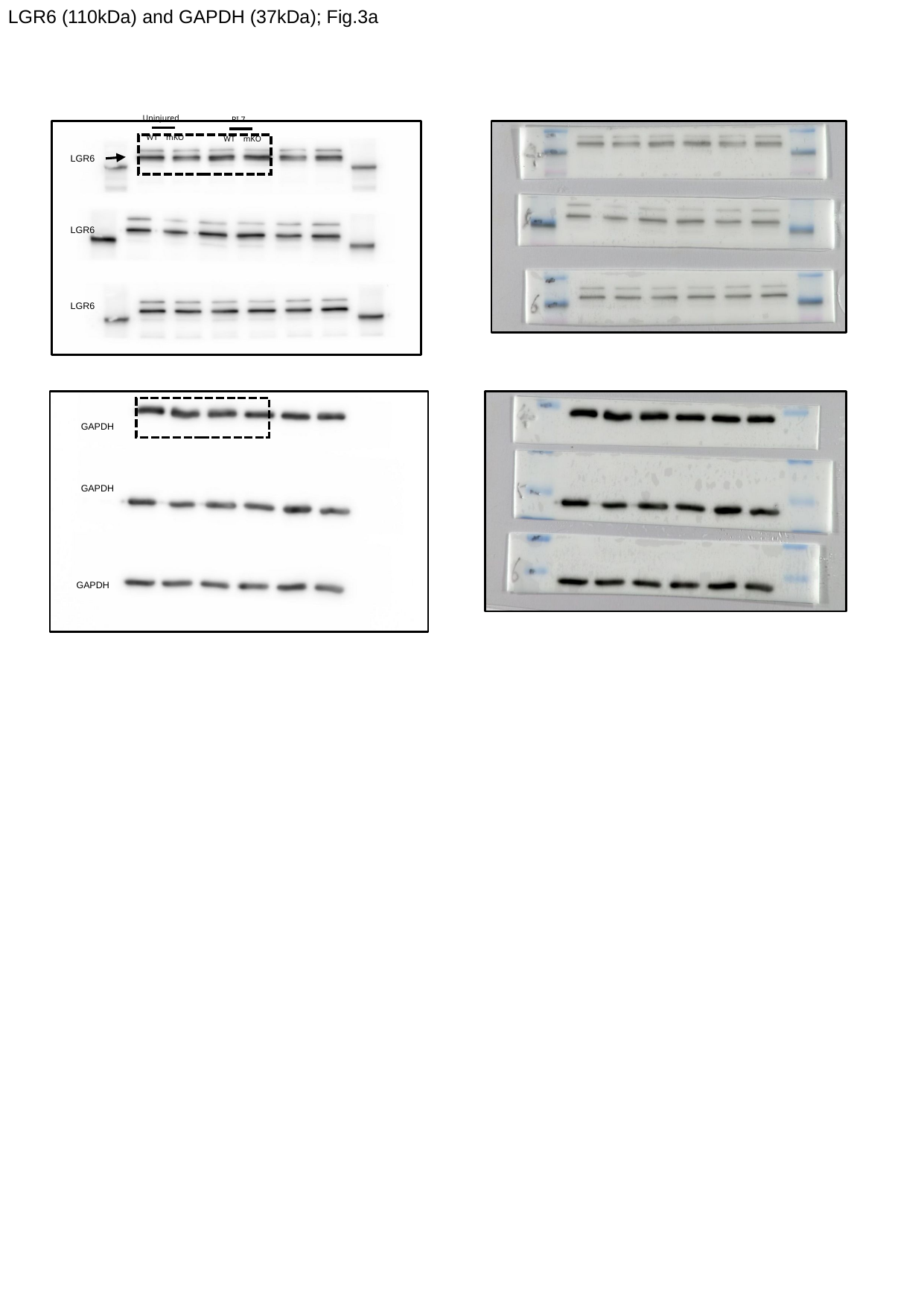

LGR6 (110kDa) and GAPDH (37kDa); Fig.3a
Uninjured
mKO
WT
PI 7
WT
mKO
LGR6
LGR6
LGR6
GAPDH
GAPDH
GAPDH

## Slide 11
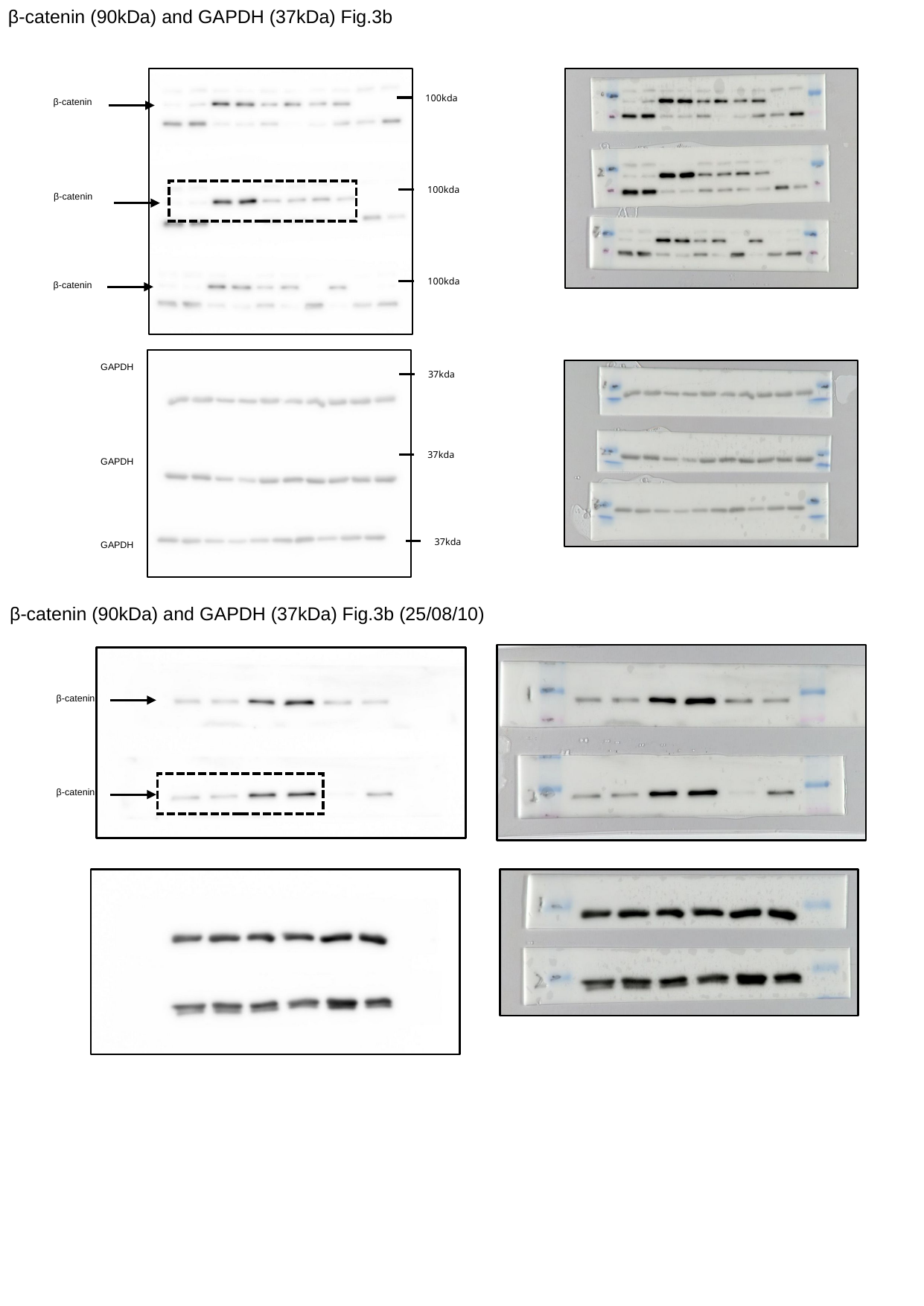

β-catenin (90kDa) and GAPDH (37kDa) Fig.3b
100kda
β-catenin
100kda
β-catenin
100kda
β-catenin
GAPDH
37kda
37kda
GAPDH
37kda
GAPDH
β-catenin (90kDa) and GAPDH (37kDa) Fig.3b (25/08/10)
β-catenin
β-catenin

## Slide 12
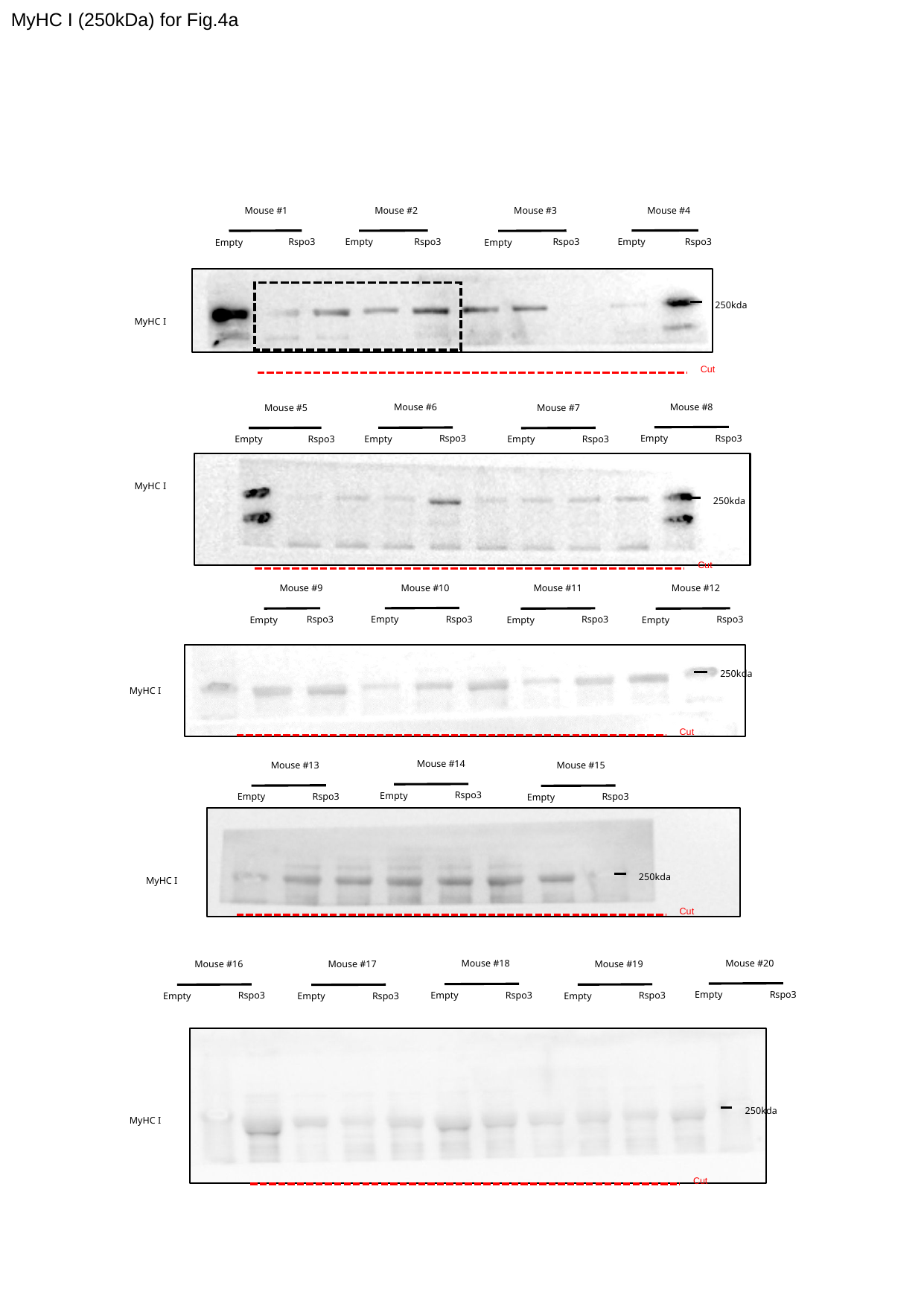

MyHC I (250kDa) for Fig.4a
Mouse #4
Rspo3
Empty
Mouse #2
Rspo3
Empty
Mouse #1
Rspo3
Empty
Mouse #3
Rspo3
Empty
250kda
MyHC I
Cut
Mouse #8
Rspo3
Empty
Mouse #6
Rspo3
Empty
Mouse #5
Rspo3
Empty
Mouse #7
Rspo3
Empty
250kda
MyHC I
Cut
Mouse #10
Rspo3
Empty
Mouse #9
Rspo3
Empty
Mouse #11
Rspo3
Empty
Mouse #12
Rspo3
Empty
250kda
MyHC I
Cut
Mouse #14
Rspo3
Empty
Mouse #13
Rspo3
Empty
Mouse #15
Rspo3
Empty
250kda
MyHC I
Cut
Mouse #20
Rspo3
Empty
Mouse #18
Rspo3
Empty
Mouse #19
Rspo3
Empty
Mouse #16
Rspo3
Empty
Mouse #17
Rspo3
Empty
250kda
MyHC I
Cut
20/12/23
21/02/06
21/03/17
21/04/28
21/06/10

## Slide 13
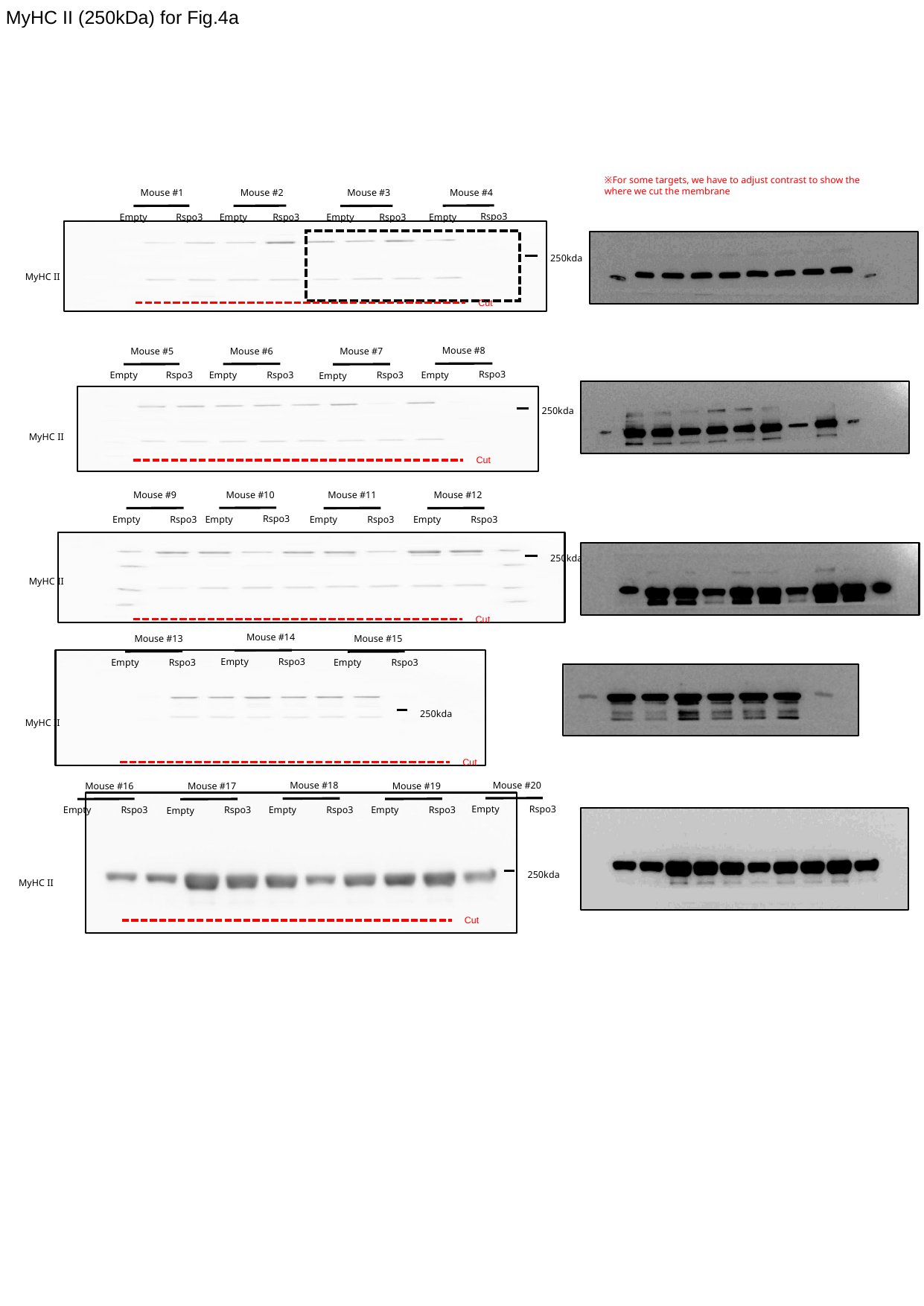

MyHC II (250kDa) for Fig.4a
※For some targets, we have to adjust contrast to show the where we cut the membrane
Mouse #4
Rspo3
Empty
Mouse #2
Rspo3
Empty
Mouse #1
Rspo3
Empty
Mouse #3
Rspo3
Empty
250kda
MyHC II
Cut
Mouse #8
Rspo3
Empty
Mouse #6
Rspo3
Empty
Mouse #5
Rspo3
Empty
Mouse #7
Rspo3
Empty
250kda
MyHC II
Cut
Mouse #10
Rspo3
Empty
Mouse #9
Rspo3
Empty
Mouse #11
Rspo3
Empty
Mouse #12
Rspo3
Empty
250kda
MyHC II
Cut
Mouse #14
Rspo3
Empty
Mouse #13
Rspo3
Empty
Mouse #15
Rspo3
Empty
250kda
MyHC II
Cut
Mouse #20
Rspo3
Empty
Mouse #18
Rspo3
Empty
Mouse #19
Rspo3
Empty
Mouse #16
Rspo3
Empty
Mouse #17
Rspo3
Empty
250kda
MyHC II
Cut

## Slide 14
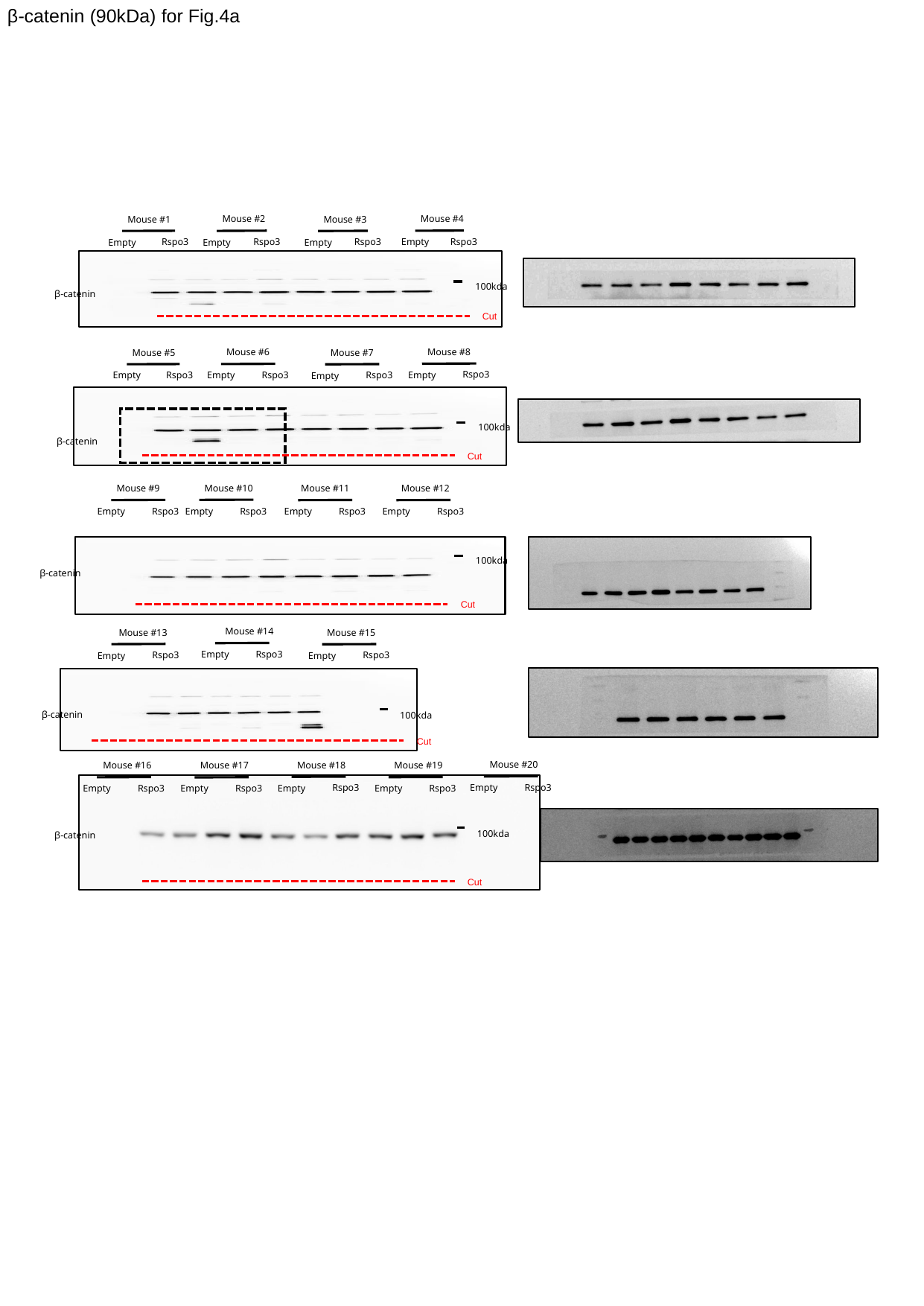

β-catenin (90kDa) for Fig.4a
Mouse #4
Rspo3
Empty
Mouse #2
Rspo3
Empty
Mouse #1
Rspo3
Empty
Mouse #3
Rspo3
Empty
100kda
β-catenin
Cut
Mouse #8
Rspo3
Empty
Mouse #6
Rspo3
Empty
Mouse #5
Rspo3
Empty
Mouse #7
Rspo3
Empty
100kda
β-catenin
Cut
Mouse #10
Rspo3
Empty
Mouse #9
Rspo3
Empty
Mouse #11
Rspo3
Empty
Mouse #12
Rspo3
Empty
100kda
β-catenin
Cut
Mouse #14
Rspo3
Empty
Mouse #13
Rspo3
Empty
Mouse #15
Rspo3
Empty
100kda
β-catenin
Cut
Mouse #20
Rspo3
Empty
Mouse #18
Rspo3
Empty
Mouse #19
Rspo3
Empty
Mouse #16
Rspo3
Empty
Mouse #17
Rspo3
Empty
100kda
β-catenin
Cut

## Slide 15
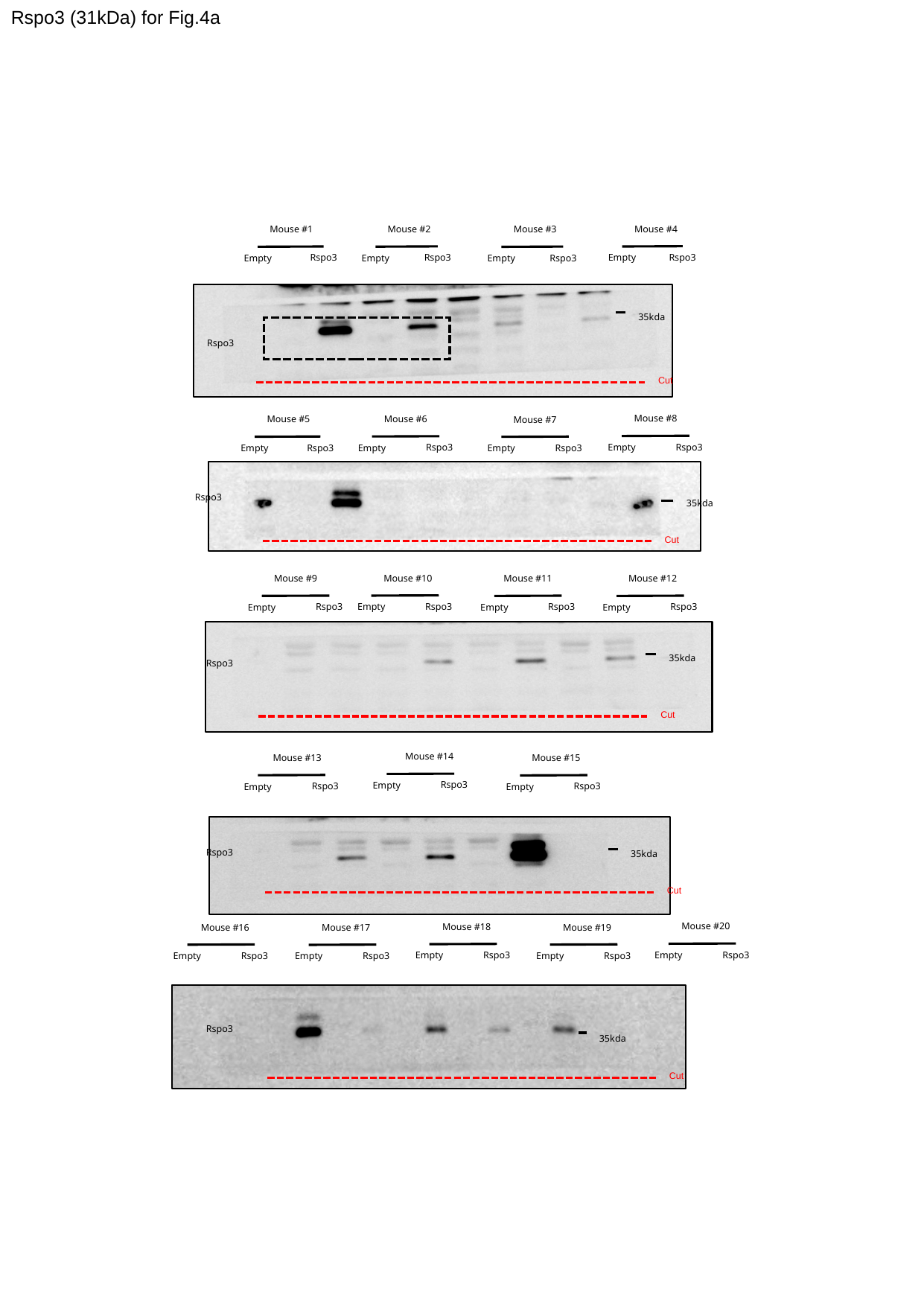

Rspo3 (31kDa) for Fig.4a
Mouse #4
Rspo3
Empty
Mouse #2
Rspo3
Empty
Mouse #1
Rspo3
Empty
Mouse #3
Rspo3
Empty
35kda
Rspo3
Cut
Mouse #8
Rspo3
Empty
Mouse #6
Rspo3
Empty
Mouse #5
Rspo3
Empty
Mouse #7
Rspo3
Empty
35kda
Rspo3
Cut
Mouse #10
Rspo3
Empty
Mouse #9
Rspo3
Empty
Mouse #11
Rspo3
Empty
Mouse #12
Rspo3
Empty
35kda
Rspo3
Cut
Mouse #14
Rspo3
Empty
Mouse #13
Rspo3
Empty
Mouse #15
Rspo3
Empty
35kda
Rspo3
Cut
Mouse #20
Rspo3
Empty
Mouse #18
Rspo3
Empty
Mouse #19
Rspo3
Empty
Mouse #16
Rspo3
Empty
Mouse #17
Rspo3
Empty
35kda
Rspo3
Cut

## Slide 16
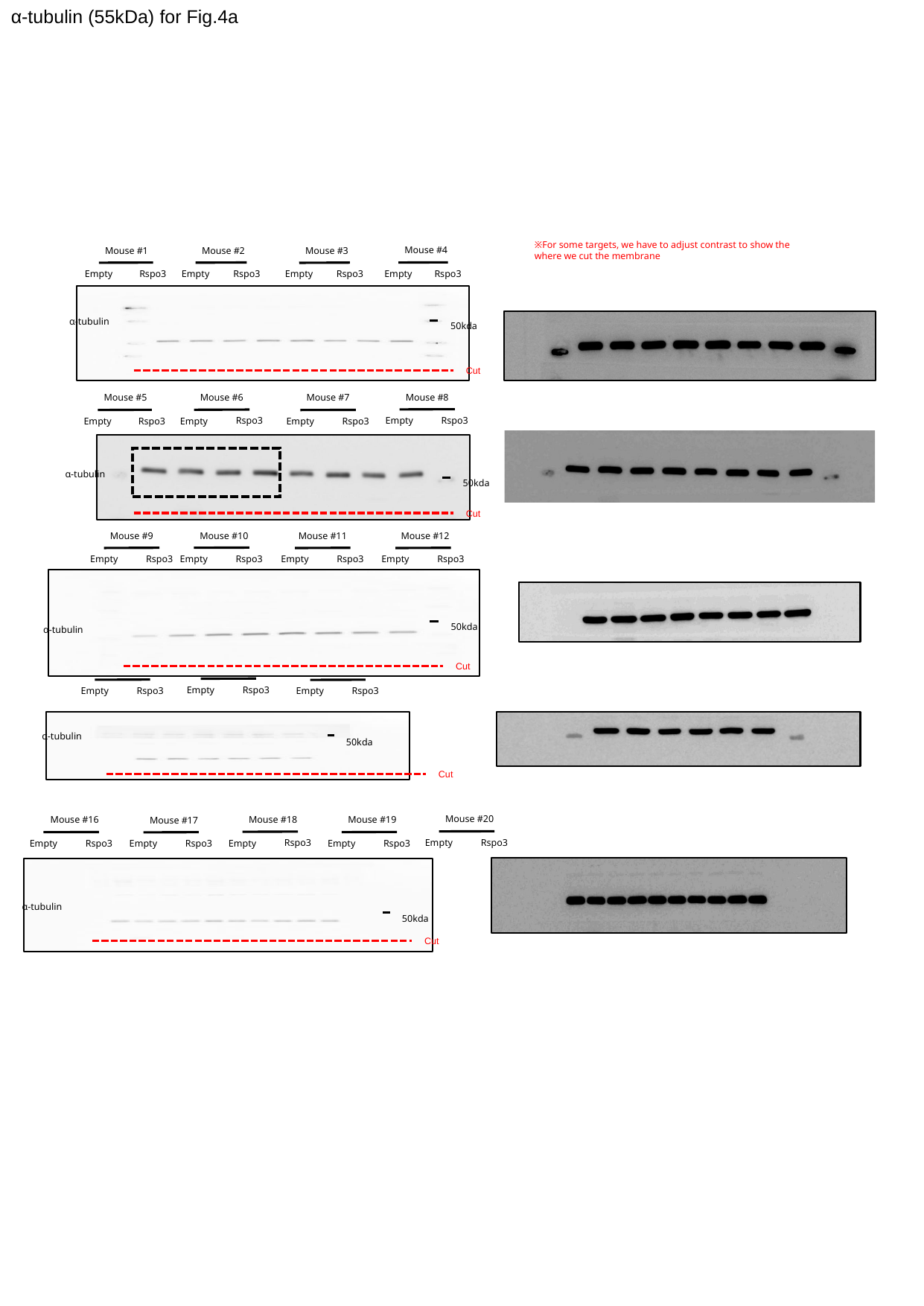

α-tubulin (55kDa) for Fig.4a
※For some targets, we have to adjust contrast to show the where we cut the membrane
Mouse #4
Rspo3
Empty
Mouse #2
Rspo3
Empty
Mouse #1
Rspo3
Empty
Mouse #3
Rspo3
Empty
50kda
α-tubulin
Cut
Mouse #8
Rspo3
Empty
Mouse #6
Rspo3
Empty
Mouse #5
Rspo3
Empty
Mouse #7
Rspo3
Empty
50kda
α-tubulin
Cut
Mouse #10
Rspo3
Empty
Mouse #9
Rspo3
Empty
Mouse #11
Rspo3
Empty
Mouse #12
Rspo3
Empty
50kda
α-tubulin
Mouse #14
Rspo3
Empty
Cut
Mouse #13
Rspo3
Empty
Mouse #15
Rspo3
Empty
50kda
α-tubulin
Cut
Mouse #20
Rspo3
Empty
Mouse #18
Rspo3
Empty
Mouse #19
Rspo3
Empty
Mouse #16
Rspo3
Empty
Mouse #17
Rspo3
Empty
50kda
α-tubulin
Cut
